# Climate‐Driven Distribution Modeling of the Glacial Apollo Butterfly (*Parnassius glacialis*) for Biodiversity Conservation

**DOI:** 10.1002/ece3.74412

**Published:** 2026-11-01

**Authors:** Yongli Wang, Mingsheng Yang, Yupei Hu, Raul Narciso C. Guedes, Youssef Dewer, Suqin Shang, Xiushan Li

**Affiliations:** ^1^ Biocontrol Engineering Laboratory of Crop Diseases and Pests of Gansu Province, College of Plant Protection Gansu Agricultural University Lanzhou Gansu China; ^2^ College of Life Science and Agronomy Zhoukou Normal University Zhoukou Henan China; ^3^ Dancheng Green Agriculture Observation and Research Station of Henan Province Zhoukou Normal University Zhoukou Henan China; ^4^ Department of Entomology Universidade Federal de Vicosa Vicosa Minas Gerais Brazil; ^5^ Department of Phytotoxicity Research, Central Agricultural Pesticide Laboratory Agricultural Research Center Giza Dokki Egypt; ^6^ Department of Entomology, College of Life Science China West Normal University Nanchong China

**Keywords:** biodiversity conservation, climate change, environmental adaptability, *Parnassius glacialis*, species distribution modeling

## Abstract

Understanding how climate change affects insect distribution is critical for biodiversity conservation, particularly in the case of range‐restricted and ecologically sensitive species. The glacial Apollo, *Parnassius glacialis* (Lepidoptera: Papilionidae), is a high‐altitude butterfly of notable scientific and esthetic value, yet its ecological response to climate change remains poorly understood. Here, we model the current and future distribution of *P. glacialis* across China and identify key environmental drivers shaping its habitat. We developed an ensemble species distribution model using 45 occurrence records and 10 bioclimatic variables, integrating outputs from multiple algorithms to enhance predictive robustness. Temperature—particularly seasonal variation—emerged as the strongest determinant of habitat suitability, followed by precipitation. At present, suitable habitats are concentrated in northern, southwestern, and eastern China. However, projections under future climate scenarios reveal a marked reduction in suitable range, with the habitat centroid shifting to lower elevations in Hubei Province. The direction and extent of this shift vary among scenarios, highlighting the species' vulnerability to both climatic and socioeconomic trajectories. These findings raise urgent conservation concerns, suggesting that *P. glacialis* may face significant habitat loss and range contraction under projected climate conditions. We recommend the establishment of targeted protected areas, the integration of insect responses into climate adaptation policies, and the implementation of long‐term ecological monitoring. This study provides crucial insights into climate‐driven range shifts in high‐altitude butterflies and underscores the broader need for adaptive conservation strategies in a warming world.

## Graphical abstract

This study addresses a pressing issue in conservation science: the vulnerability of high‐altitude, range‐restricted insect species to climate change. The Glacial Apollo (*Parnassius glacialis*) is a butterfly of significant ecological and esthetic value, yet little is known about its response to projected climate scenarios. Using an ensemble species distribution modeling approach, our work identifies the current and future suitable habitats for *P. glacialis* , reveals key environmental drivers (notably temperature seasonality), and forecasts distributional shifts under multiple Shared Socioeconomic Pathway scenarios. These results carry important implications for developing targeted conservation strategies and climate‐adaptive management policies in mountainous ecosystems.

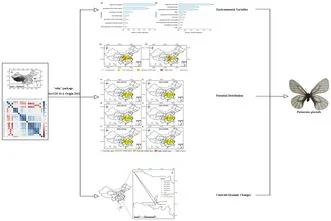

## 1 Introduction

Human activities and climate change are major drivers of species' spatiotemporal shifts, often resulting in habitat fragmentation and ecosystem instability (Li et al. 2024; Yang, Gu, and Wang 2024; Yang, Huo, et al. 2024; Feng et al. 2024; Gudko et al. 2024). This rapid transformation has contributed to a global decline in biodiversity, as species face unprecedented challenges to adapt (Paterson et al. 2024). Rising temperatures, in particular, are forcing species to shift their suitable habitats toward higher elevations and latitudes (Gao et al. 2024), compounding the impacts of habitat loss and fragmentation.

Butterflies, due to their sensitivity to environmental changes, serve as effective bioindicators for monitoring ecosystem health (Parmesan et al. 1999; Devictor et al. 2012). Beyond their ecological roles, butterflies are also important to agriculture, forestry, and environmental conservation, and they hold significant cultural and esthetic value (Fang et al. 2023; Rivest and Kharouba 2024; Halsch et al. 2024). China, renowned for its exceptional biodiversity, harbors 2153 butterfly species. However, this rich diversity is increasingly threatened by rapid industrialization and habitat alteration (Zhang, Wang, et al. 2022; Zhang, Zou, et al. 2022; Dong et al. 2024; Wang and Wang 2024). Among China's butterfly fauna, the genus *Parnassius* (subfamily Parnassiinae), known for its high‐altitude and often rare species, comprises approximately 60 species distributed across Central Asia, the Himalayas, western China, and Japan (Schmitt 2007; Condamine 2018).

*Parnassius* species, which typically oviposit on rocky surfaces and dead branches, are well adapted to cold environments. One of their key physiological adaptations is the darkening of wing bases at higher altitudes, which enables greater heat absorption and enhances survival in colder habitats (Ackery 1975; Su et al. 2020). Despite these adaptations, habitat loss and climate change have placed several *Parnassius* species at risk, with many now listed on the IUCN Red List and China's List of Wildlife under State Protection (https://www.gov.cn/xinwen/2021) (Todisco et al. 2010). Among them, the Glacial Apollo, *Parnassius glacialis*, is a species of high‐altitude origin that has uniquely colonized low‐elevation habitats in central‐eastern China (Zhao et al. 2023). It displays notable ecological flexibility, occupying both montane and lower‐elevation regions, including parts of southeastern China (Zhou 1999; Wang et al. 2019).

*P. glacialis* is univoltine. It oviposits on stones or withered branches from May to June (Figure S1a). The overwintering eggs hatch into larvae in February to March of the following year. Larvae pupate in April, and adults eclose around May. The larvae feed on *Corydalis yanhusuo* and *Corydalis edulis*, and the adult butterflies primarily utilize *Rubus hirsutus* and *Robinia pseudoacacia* as nectar sources (Wu 1998a, 1998b; Awata et al. 2010). It mainly inhabits low‐elevation forest edges, roadsides, and forest openings, preferring sunny and dry habitats. As the only *Parnassius* species distributed in low‐elevation areas of the Jiangnan region, it possesses important ecological significance, including value as evolutionary research material, ornamental worth, and role as an environmental indicator species (Wu 1998a, 1998b; Wang et al. 2019). Characterized by its distinct white wings and a black longitudinal band, *P. glacialis* has primarily been studied in terms of its morphology, phylogeography, and genomics (Akiyama and Nishida 2013; Hao et al. 2017; Tao et al. 2020; Si et al. 2020; Hu et al. 2022; Su et al. 2023). However, its potential distribution and biogeographical dynamics remain underexplored.

Ecological Niche Models (ENMs), also termed Species Distribution Models (SDMs), provide a robust analytical framework for exploring species' potential habitats and their ecological responses to climate change. By integrating species occurrence records and multi‐source environmental variables, ENMs reliably predict spatial distribution patterns and habitat suitability (Elith and Leathwick 2009; Neupane et al. 2024). Spatiotemporal dynamic assessments based on ENMs help clarify climate‐induced biodiversity variations and support targeted conservation strategy formulation (Mammola et al. 2021). Classical modeling algorithms, including Generalized Linear Models (GLM), Maximum Entropy (MaxEnt), and Random Forest (RF), have been extensively applied in SDM research (Marmion et al. 2009; Newbold et al. 2009). Notably, MaxEnt exhibits excellent performance for species with limited occurrence data (Volkov et al. 2009). Compared with single‐algorithm models, ensemble models synthesize multi‐model predictions, effectively improving predictive accuracy and reducing simulation uncertainty (Soberon and Peterson 2005; Elith and Leathwick 2009; Thuiller et al. 2009; Liu et al. 2020; Gong et al. 2020).

In this study, we adopted an ensemble modeling framework to predict the current and future potential habitats of *P. glacialis* . By incorporating multiple driving factors, including climate, elevation, human population density (HPD), and Normalized Difference Vegetation Index (NDVI), we systematically explored the distribution dynamics of *P. glacialis* under changing environmental conditions. This study aims to clarify how climate change reshapes the biogeographic range of *P. glacialis* and provide scientific references for the long‐term conservation and adaptive management of this vulnerable butterfly species.

## 2 Materials and Methods

### 2.1 Species Distribution Data

Currently, the Global Biodiversity Information Facility (GBIF) database has been widely used (Yu et al. 2023; Li et al. 2024), and studies have shown that combining species occurrence records from published literature or known species distribution points from species databases with environmental variables allows for the modeling of species' distribution ranges under current or future climate conditions (Rong et al. 2019; Khan et al. 2022). Models constructed using species occurrence records distributed within the study area can yield accurate prediction results (Li et al. 2024; Ran et al. 2024).

We compiled national occurrence records of *P. glacialis* from two sources: peer‐reviewed published literature (Wang et al. 2019; Su 2019) and the GBIF (https://www.gbif.org, accessed May 2023, dataset DOI: https://doi.org/10.15468/dl.xkq94z). As a global open biodiversity database, GBIF supports flexible retrieval of either global or geographically restricted species occurrence datasets tailored to research objectives. The standardized workflow for acquiring and preprocessing GBIF data in this study was as follows: (1) Input the scientific name of *P. glacialis* on the GBIF homepage, then filter and enter the occurrence record retrieval page; (2) Extract valid occurrence records exclusively belonging to the target species; (3) Constrain the geographic scope to China to implement spatial screening and preliminary data sorting; (4) Export the filtered dataset of species distribution points; (5) Conduct data cleaning to eliminate invalid records, and the cleaned dataset was adopted as raw occurrence data for subsequent modeling.

For literature‐derived records, to maintain the highest possible spatial accuracy and eliminate the inherent spatial uncertainty associated with descriptive locality names (e.g., city‐level names), we strictly excluded all records that lacked precise geographic coordinates. Only records where exact longitude and latitude were directly provided in the original publications or their Supporting Information were retained.

In total, 47 raw occurrence records were collected: 45 from GBIF and 2 from published literature (Table S1). To enhance data quality, we used the R package “CoordinateCleaner” (Zizka et al. 2019) to eliminate duplicates and erroneous records (e.g., points located in the ocean or administrative centers). Spatial bias was reduced using the “spThin” package (Aiello‐Lammens et al. 2015) with a fixed thinning distance of 10 km, ensuring that no more than one record was retained per 10 × 10 km environmental grid cell.

After filtering, 45 occurrence points remained for modeling (Figure S1b and Table S2).

### 2.2 Environmental Variables

At broad geographical scales, species distributions are primarily influenced by climate and altitude, particularly in relation to temperature gradients (Hof et al. 2012; Urbani et al. 2017). Two mainstream climate datasets are widely applied in bioclimatic modeling: CHELSA performs better for regions with rugged terrain or research requiring high‐precision precipitation data, while WorldClim is a mature, open‐access database of fundamental climate layers. Considering the ecological traits of *P. glacialis* , we extracted 19 bioclimatic variables and one elevation layer from WorldClim v2.1 (http://www.worldclim.org/) at a spatial resolution of 2.5 arc‐minutes. This dataset contains baseline historical climate records (1970–2000) and future climate projections spanning multiple periods.

Compared with CMIP5, the sixth phase of the Coupled Model Intercomparison Project (CMIP6) delivers enhanced climate simulations and more robust representation of interactions between socioeconomic development and climate change (Moss et al. 2010; Harris et al. 2014; Fick and Hijmans 2017; Fan et al. 2020). To alleviate uncertainties originating from inter‐model discrepancies and comply with standard analytical frameworks for insect species distribution modeling (Yang, Gu, and Wang 2024; Yang, Huo, et al. 2024; Liu et al. 2025), we adopted three frequently used CMIP6 Global Climate Models (GCMs): BCC‐CSM2‐MR, IPSL‐CM6A‐LR, and MRI‐ESM2‐0. WorldClim provides four Shared Socioeconomic Pathways (SSPs): SSP1‐2.6 (low emissions), SSP2‐4.5 (moderate emissions), SSP3‐7.0 (medium‐high emissions), and SSP5‐8.5 (high emissions) (Fischer et al. 2005). We selected SSP1‐2.6 and SSP5‐8.5 to characterize the lowest and highest greenhouse gas emission pathways for projecting the potential distribution of *P. glacialis* across China.

The selection of BCC‐CSM2‐MR, IPSL‐CM6A‐LR, and MRI‐ESM2‐0 was based on objective criteria: (1) their demonstrated high skill in reproducing observed precipitation and temperature patterns over the East Asian monsoon region (including China), as validated in previous regional climate model evaluation studies; (2) their competency in simulating the complex topographical climatic interactions typical of the central‐eastern Chinese landscape; and (3) their distinct equilibrium climate sensitivities, which together encompass a broad range of plausible future warming pathways (Jiang et al. 2020; Wang et al. 2025; Scafetta 2023).

For each GCM, we included the two SSPs across three future time slices: the 2030s (2021–2040), 2050s (2041–2060), and 2070s (2061–2080), generating a total of 18 distinct climate scenarios: BCC‐CSM2‐MR_ssp126_2021–2040, BCC‐CSM2‐MR_ssp585_2021–2040, BCC‐CSM2‐MR_ssp126_2041–2060, BCC‐CSM2‐MR_ssp585_2041–2060, BCC‐CSM2‐MR_ssp126_2061–2080, BCC‐CSM2‐MR_ssp585_2061–2080, IPSL‐CM6A‐LR_ssp126_2021–2040, IPSL‐CM6A‐LR_ssp585_2021–2040, IPSL‐CM6A‐LR_ssp126_2041–2060, IPSL‐CM6A‐LR_ssp585_2041–2060, IPSL‐CM6A‐LR_ssp126_2061–2080, IPSL‐CM6A‐LR_ssp585_2061–2080, MRI‐ESM2‐0_ssp126_2021–2040, MRI‐ESM2‐0_ssp585_2021–2040, MRI‐ESM2‐0_ssp126_2041–2060, MRI‐ESM2‐0_ssp585_2041–2060, MRI‐ESM2‐0_ssp126_2061–2080, MRI‐ESM2‐0_ssp585_2061–2080.

To mitigate multicollinearity between environmental predictors, we first standardized climate variables via the “sdm” package in R (Figure S2b). Subsequently, Pearson correlation analysis (Figure S2a) was performed using the “corrplot” package to screen bioclimatic variables for subsequent modeling. Specifically, pairwise Pearson correlation coefficients (*r*) were calculated, and any variable pair with a strong correlation (|*r*| ≥ 0.8) was pruned by randomly removing one variable from each correlated pair to lower the risk of model overfitting (McKenna et al. 2016).

In addition to climate and elevation data, we included two commonly used proxies of anthropogenic disturbance: human population density (HPD) and human footprint index (HFP). For HPD: data year 2000, spatial resolution of 0.008333 decimal degrees (approximately 0.93 km at the equator), dataset version Gridded Population of the World Version 4 (GPWv4), Cartesian coordinate system, global coverage, data supported by the Earth Science Data Information System (ESDIS), available at http://sedac.ciesin.columbia.edu. For HFP: data year 2018, spatial resolution of 0.008333 decimal degrees, dataset version Last of the Wild Project Version 2 (LWP‐2), Cartesian coordinate system, global coverage, data supported by the Earth Science Data Information System (ESDIS), available at https://www.earthdata.nasa.gov/. It should be noted that the non‐climatic variables (HPD, NDVI) represent contemporary static environmental constraints on species distribution rather than temporally dynamic variables. These datasets were employed exclusively for characterizing current habitat conditions (1970–2000 climate baseline matched with contemporary HPD and NDVI), as they are not used in future climate scenario projections (see above). Although the data years differ, this temporal mismatch is unlikely to affect our future projections because only climatic variables were used for future predictions. Nevertheless, we acknowledge that the different temporal origins of these datasets represent a limitation of the current study when interpreting contemporary habitat suitability. These two anthropogenic variables exhibited strong correlation (|*r*| > 0.8; Figure S3b), so only HPD was retained for model construction.

We also incorporated the Normalized Difference Vegetation Index (NDVI): data year 2023, spatial resolution of 250 m, dataset version MOD13Q1 Version 6, Cartesian coordinate system. The NDVI data were captured by the PFM sensor aboard the Terra satellite and the FM1 MODIS sensor aboard the Aqua satellite, based on the MODIS Sinusoidal Tiling System, with the data source at https://lpdaac.usgs.gov/products/mod13q1v061/. NDVI remote sensing data were processed using MRT 4.1 (NASA, USA), ENVI 5.3 (Exelis Visual Information Solutions Corporation, USA), and ArcGIS 10.4 (Esri, Redlands, California, USA), with monthly values aggregated into annual means. Since the spatial resolutions of the environmental variables differed, we standardized them using the nearest‐neighbor resampling method via the R package “raster” (Hijmans et al. 2015) with the nearest‐neighbor resampling method to standardize the resolution across all datasets.

Nearest‐neighbor resampling was selected because (i) it preserves the original pixel values of continuous environmental variables without introducing artificial values through interpolation, thereby maintaining data integrity; (ii) it better preserves environmental heterogeneity compared to bilinear or cubic convolution methods; and (iii) given that all variables were resampled to a coarser target resolution (2.5 arc‐minutes, ~4.5 km), which is substantially larger than the original resolutions (250 m to ~1 km), the aggregation effect within each target pixel smooths local sampling variation. This approach is widely adopted in species distribution modeling when standardizing environmental layers of varying resolutions (Hijmans and Graham 2006; Beck et al. 2014).

After preprocessing and collinearity screening (Figure S2), a total of 10 environmental variables were selected for model construction (Figure S3a and Table S3). In our model projections under climate change scenarios, only climatic variables were employed, with no other variables included. Crucially, all temporal comparisons between current and future distributions (Figures 5, 6, 7) are based exclusively on this climate‐only model, ensuring that observed shifts reflect climate‐driven changes rather than artifacts of changing predictor composition. The full‐variable model (including climate, elevation, HPD, and NDVI) was constructed solely for the purpose of characterizing the current potential distribution with additional ecological constraints (Figure 4b) and was not used in any comparison with future projections.

### 2.3 Model Development

The calibration area (CA), also termed the accessible area of target species, refers to the geographic extent and corresponding environmental space used for training, fitting, parameter optimization, and validation of correlative species distribution models. It delineates the range within which the focal species could disperse, colonize, and establish viable populations under recent biogeographic conditions. By constraining model training within ecologically reasonable accessible space, the CA eliminates bias from meaningless background points sampled beyond the species' potential dispersal scope and enables the model to accurately capture the species' realized ecological niche (Rojas‐Soto et al. 2024).

Among existing delimitation approaches for calibration areas, the Minimum Convex Polygon (MCP) method combined with outward buffer zones is the most widely adopted protocol for insect distribution modeling (Baloch et al. 2020). In this study, ArcGIS 10.4 was applied to generate the final calibration area for *P. glacialis* , following a standardized workflow: (1) Prepare cleaned, geographically filtered occurrence records of *P. glacialis* ; (2) Generate the MCP polygon enclosing all valid presence points via the Minimum Bounding Geometry tool in the Data Management Toolbox; (3) Create a 50‐km outward buffer zone based on the MCP polygon using the Buffer tool in the Analysis Toolbox, which served as the definitive calibration boundary; (4) Unify the coordinate projection of all environmental raster layers, then clip each layer using the final calibration polygon as the mask.

It should be noted that this calibration area delineation strategy does not rely on external hierarchical biogeographic classifications (e.g., ecoregions or watershed units), but rather adopts a geometrically defined accessible space derived from the MCP extent of all validated occurrence points expanded by a 50‐km buffer. The selection of the 50‐km buffer distance is grounded in two considerations: (i) empirical precedents from mark‐recapture studies on congeneric high‐altitude *Parnassius* species, which report effective dispersal radii generally within 20–30 km—the 50‐km buffer thus conservatively encompasses undetected satellite populations while avoiding over‐extension into lowland unsuitable habitats; and (ii) topographic autocorrelation—given the steep elevational gradients in the study region, the 50‐km expansion effectively follows contiguous high‐elevation ridgelines and valleys that serve as natural dispersal corridors, rather than crossing deeply incised river gorges that act as barriers. Specifically, the 50‐km buffer was grounded in topographical autocorrelation—given the steep elevational gradients in the study region, the buffer effectively follows the mountain chains rather than crossing the deep valleys that serve as barriers, thereby defining a topographically accessible area. To ensure full transparency and to visually validate the ecological logic of our CA, the final calibration boundary and its overlaid occurrence records are presented in Figure S4. The map utilizes an overview‐zoom layout: the inset map shows the national boundaries, while the main panel focuses on central‐eastern China, incorporating a semi‐transparent Hillshade layer. As shown, the 50‐km buffer is not a purely geometric shape; it naturally follows contiguous high‐elevation ridgelines (e.g., the Qinling‐Daba Mountains) that act as dispersal corridors, while avoiding deeply incised river gorges. This clearly identifies the specific biogeographic entities encompassed within the CA and demonstrates that this area constitutes the realistic accessible region for *P. glacialis* .

We used the R package “sdm” (Naimi and Araujo 2016) to develop species distribution models (SDMs) by integrating occurrence data and environmental predictors. Twelve widely used modeling algorithms were employed: MaxEnt (Phillips et al. 2006), RF (Breiman 2001), Generalized Additive Model (GAM) (Hastie and Tibshirani 1987), GLM (Nelder and Wedderburn 1972), Classification and Regression Tree Model (CART) (Loh 2011), Flexible Discriminant Analysis (FDA) (Hastie et al. 1994), Multivariable Adaptive Regression Spline (MARS) (Friedman 1991), Support Vector Machine (SVM) (Hearst et al. 1998), MaxLike (Royle et al. 2012), Domain (Reinhartz‐Berger 2010), GLMNET (Engebretsen and Bohlin 2019), and Multivariate Discriminant Analysis (MDA) (Choubin et al. 2019).

To strengthen model reliability, we generated pseudo‐absence and background points using the “sdmData” function, with distinct strategies tailored to different modeling algorithms. Specifically, among the 12 algorithms employed: (i) MaxEnt and MaxLike operate under a presence‐background framework and thus used only 10,000 background points; (ii) the remaining 10 algorithms (RF, GAM, GLM, CART, FDA, MARS, SVM, Domain, GLMNET, and MDA) operate under a presence‐pseudoabsence framework and used 1000 pseudo‐absence points. Both pseudo‐absence and background points were randomly sampled within the delineated calibration area (CA) without applying exclusion buffers around occurrence records. The decision to omit exclusion buffers was based on: (a) the relatively small CA extent (MCP plus 50‐km buffer), where imposing buffers could artificially exclude environmentally similar background space and lead to conservative niche boundary estimation; (b) prior application of spThin (10‐km thinning distance) already reduced spatial autocorrelation among occurrence points; and (c) recommendations from Barbet‐Massin et al. (2012) that random sampling without buffers often achieves a favorable balance between bias and variance under small sample sizes (*n* < 50). The generated point sets were kept fixed across all 10 repeated random sub‐sampling (75:25 holdout) runs to ensure fair comparison among algorithms, with only the training (75%) and testing (25%) data partitions varying across iterations. A summary of the pseudo‐absence/background point configurations for each algorithm is provided in Table S4.

All 12 algorithms were implemented using the default hyperparameter configurations provided by the ‘sdm’ package (Naimi and Araujo 2016), without additional systematic tuning. The key hyperparameter settings are summarized in Table S5. For MaxEnt, the regularization multiplier was set to 1 (default), feature classes were automatically selected from linear, quadratic, product, threshold, and hinge features, with a convergence threshold of 1 × 10^−5^ and maximum iterations of 1000. For RF, the number of trees (ntree) was 500, with mtry set to the square root of the number of predictor variables (default). GAM employed penalized splines with a basis dimension (k) of 10 (default). SVM used a radial basis kernel with cost = 1 and gamma = 1/nvars (default). MARS used an interaction degree of 1 (additive model only) with penalty = 2 and a maximum basis function count of 21. GLMNET employed an elastic‐net mixing parameter α = 1 (LASSO default), with the regularization parameter λ automatically selected via built‐in cross‐validation. The remaining algorithms (GLM, CART, FDA, Domain, and MDA) were run with their respective R default parameters, as detailed in Table S5.

We acknowledge that no systematic hyperparameter tuning (e.g., grid search or Bayesian optimization) was performed in this study. This decision was based on three considerations: (i) with only 45 occurrence records (a small‐sample scenario), further partitioning the data into separate tuning, training, and testing sets would substantially reduce training set representativeness and exacerbate overfitting risks; (ii) the default parameters in the ‘sdm’ package have been validated across a wide range of ecological modeling applications and generally provide stable and reasonable performance; (iii) the final ensemble model, which employs a weighted averaging strategy across multiple algorithms, can effectively buffer the uncertainty introduced by hyperparameter choices in individual algorithms (Hao et al. 2019; Marmion et al. 2009).

Model performance was assessed using 10 repeated random sub‐sampling (75:25 holdout) runs (Naimi and Araujo 2016), with a maximum number of iterations of 5000 to ensure convergence (Wang et al. 2020; Liu et al. 2022; Zhang, Wang, et al. 2022; Zhang, Zou, et al. 2022). In total, 120 individual models were constructed. Individual model performance was evaluated based on the Receiver Operating Characteristic (ROC) curve and True Skill Statistic (TSS), using thresholds of AUC ≥ 0.95 and TSS ≥ 0.85 for model inclusion. Models meeting these criteria were integrated into an ensemble using the ensemble function (Naimi and Araujo 2016), which employs a weighted averaging strategy to enhance predictive accuracy (Hao et al. 2019).

To examine model performance, we generated ROC and response curves for each environmental variable using the “roc” and “curve” functions, respectively. These were visualized using the “ggplot2” package (Snow 2011). To quantify the relative importance of each environmental variable during model construction, we followed the analytical workflow below. First, the “getVarImp” function was applied to extract importance values for all environmental predictors. Next, these values were sorted in descending order. Finally, Origin 2021 was used for data visualization. Variable importance was quantified using the ‘getVarImp’ function from the ‘sdm’ package. The underlying calculation of importance differs by algorithm: for MaxEnt, importance is based on permutation importance, measured as the decrease in AUC when the variable is randomly permuted; for RF, it is based on the mean decrease in Gini impurity; for GLM, GAM, GLMNET, MARS, FDA, and MDA, it is based on standardized absolute coefficients; for SVM, it is based on coefficient weights in the hyperplane definition; for CART, it is based on the total reduction in squared error or Gini impurity at splits; and for Domain and MaxLike, it is based on the correlation difference between single‐variable and full‐variable models. Importance was calculated separately for each individual algorithm that met the inclusion criteria (AUC ≥ 0.95 and TSS ≥ 0.85) within each variable combination. For each algorithm, raw importance values from each of the 10 repeated runs were normalized to a 0–100 scale, where the most important variable was assigned a value of 100 and others were scaled proportionally. The normalized values were then averaged across the ten runs to obtain the final importance estimate for each algorithm. Finally, for each environmental variable, the importance values were averaged across all contributing algorithms to yield the combined importance (reported as percentages in Figure 2). This procedure was performed separately for the climate‐only and full‐variable model combinations, ensuring that the reported importance values are specific to each model configuration.

### 2.4 Model Evaluation and Spatial Analyses

To integrate the projections from the three GCMs, we calculated the arithmetic mean (ensemble mean) of the suitability layers for each GCM within each specific future time slice and SSP scenario using the “Cell Statistics” tool in ArcGIS 10.4. To evaluate and present inter‐model uncertainty, we further calculated the standard deviation (SD) of the predicted suitability values across the three GCMs for each time slice. These SD layers were mapped to provide a spatially explicit representation of model agreement, where high SD values denote low model consensus and correspondingly higher projection uncertainty.

We employed 10 repeated random sub‐sampling (75:25 holdout) runs to assess model accuracy. This was implemented using the replication = ’cross’ and test.percent = 25 settings in the sdm R package (Naimi and Araujo 2016), where the dataset was randomly partitioned into a 75% training set and a 25% testing set for each repetition (*n* = 10). This approach minimizes selection bias and guards against overfitting, and the generated point sets were kept fixed across all repetitions to ensure fair comparison among algorithms.

We evaluated model performance using two conventional metrics, the Area under the Curve (AUC) and True Skill Statistic (TSS). To avoid inflated evaluation results and degraded discriminatory ability induced by unreasonable calibration area size, we further supplemented the model validation with three comprehensive statistical metrics via the “ecospat” and “terra” packages in R, including the Boyce index, Sørensen similarity index, and Jaccard similarity index (Lobo et al. 2008; VanDerWal et al. 2009; Anderson and Raza 2010; Barbet‐Massin et al. 2012; Amaro et al. 2023).

The Boyce index was implemented via the ‘ecospat.boyce’ function in the ‘ecospat’ package, which evaluates the correlation between predicted suitability ranks and observed presence frequencies across 10 equally spaced bins. These three indices (Boyce, Sørensen, and Jaccard) were calculated specifically for the final ensemble model. In contrast, AUC and TSS were calculated for each of the 12 individual algorithms to enable algorithm selection for ensemble construction.

Because no observed occurrence data exist for future scenarios, the Sørensen and Jaccard indices were not calculated as validation statistics. Instead, they were calculated as map similarity indices to quantify the spatial overlap of suitable habitats between the current and future projections (e.g., current vs. 2030s SSP1‐2.6). Using the MTSPS threshold of 0.2 obtained from the ensemble model, we binarized the continuous current and future suitability rasters using the classify function in the terra R package. The indices were then computed based on the confusion matrices derived from these binary maps (TP, FP, and FN). The Jaccard index was computed as J = TP / (TP + FP + FN), and the Sørensen index as S = 2TP / (2TP + FP + FN), where TP, FP, and FN represent true positives, false positives, and false negatives, respectively.

According to the widely recognized evaluation criteria, a Boyce index above 0.7 indicates excellent model performance. Notably, the model exhibits poor predictive accuracy when the Sørensen index ≤ 0.6 or the Jaccard index ≤ 0.5. AUC is considered one of the most effective indicators for evaluating species distribution models, with a value ≥ 0.7 indicating good predictive performance (Mudereri et al. 2021). However, some limitations of AUC have been noted (Lobo et al. 2008). Therefore, we also used TSS, an improved validation metric derived from the “Kappa” coefficient. TSS value > 0.55 indicates high model accuracy, while TSS > 0.7 indicates very high accuracy (Monserud and Leemans 1992).

We extracted the AUC and TSS evaluation metrics of 12 models using the getEvaluation function, and visualized the differences in model performance through boxplots in Origin 2021 (OriginLab Corporation, Northampton, MA, USA).

For spatial pattern interpretation of species habitat suitability, we first integrated the simulation outputs from multiple GCMs by calculating the ensemble mean value in ArcGIS 10.4. Subsequently, the averaged suitability results were further classified into four grades according to standard threshold criteria: unsuitable (0.0–0.2), low suitability (0.2–0.4), moderate suitability (0.4–0.6), and high suitability (0.6–1.0) (Zhang et al. 2018; Liu et al. 2022).

To assess the conservation effectiveness of existing protected areas, we overlaid the predicted suitable habitats of *P. glacialis* with the boundaries of national nature reserves. Reserve vector data were acquired from the National Specimen Information Infrastructure (http://bhq.papc.cn/specimen.html), which enabled quantitative evaluation of the coverage of suitable habitats within protected lands and further informed targeted biodiversity conservation management strategies. Additionally, we constructed natural dispersal scenarios under multiple future climate conditions by integrating ArcGIS 10.4 and R programming, following the standardized workflow below: (1) Convert continuous suitability layers of current suitable habitats into binary presence–absence rasters using the Quick Reclassify to Binary tool in ArcGIS 10.4; (2) Generate a mask layer representing the species' extant distribution range via the “terra” and “sf” packages in R; (3) Simulate natural dispersal patterns of *P. glacialis* under each future climate scenario using terra and sf, and export corresponding dispersal raster outputs.

To analyze temporal habitat dynamics, we used the SDM_Toolbox_v2.5 plugin in ArcGIS 10.4 (Esri, Redlands, California, USA) (Brown 2014). The “Quick Reclassify to Binary” tool quantified changes in suitable over time. The threshold of maximum training sensitivity plus specificity (MTSPS) is the optimal cutoff for studies focusing on temporal habitat dynamics, habitat area comparison and centroid migration, and it is also the officially recommended standard threshold for MaxEnt models (Chen, Longzhu, et al. 2023). By contrast, the minimum training presence (MTP) threshold and percentile threshold only serve as auxiliary comparisons and are not suitable for independent quantitative analysis of habitat area shifts (Norris 2014). Numerous previous species distribution modeling studies adopted the MTSPS criterion to distinguish suitable habitats and reliably quantify habitat dynamics (Zhang et al. 2018; Bao et al. 2022; Yang, Gu, and Wang 2024; Yang, Huo, et al. 2024).

The MTSPS threshold was calculated for each individual algorithm that met the inclusion criteria (AUC ≥ 0.95 and TSS ≥ 0.85) within each variable combination, across each of the 10 repeated random sub‐sampling runs. Specifically, the ‘getThreshold’ function in the ‘sdm’ package was used to identify the threshold value that maximized the sum of sensitivity and specificity (i.e., the point on the ROC curve closest to the top‐left corner). The resulting MTSPS values were then averaged across all contributing algorithms and all runs to obtain the final single threshold of 0.2. We acknowledge that MTSPS values varied slightly across algorithms and runs, but the mean value converged to 0.20, justifying the use of a single threshold. This single threshold was applied uniformly across all GCMs and future scenarios to ensure that temporal comparisons of habitat area and centroid shifts reflect genuine climate‐driven changes rather than variations in threshold selection. It is important to distinguish between the binary threshold (MTSPS = 0.2) used for classifying suitable vs. unsuitable habitats and the suitability category thresholds (0.2, 0.4, 0.6) used for visualizing low, moderate, and high suitability grades. The binary threshold is data‐driven and statistically derived from model training performance, and it is used for all quantitative analyses of habitat area, expansion/contraction, and centroid migration. The suitability category thresholds (0.2, 0.4, 0.6) are conventional classification standards adopted from previous studies (Zhang et al. 2018; Liu et al. 2022) and serve only for visualization and ecological interpretation; they are not used in any quantitative analysis of habitat dynamics.

Accordingly, we applied the MTSPS criterion to determine a threshold value of 0.2 in this study; habitats with suitability values higher than 0.2 were classified as suitable, while those below 0.2 were regarded as unsuitable. This binary classification scheme laid a foundation for quantifying the area variation and centroid migration of potential suitable habitats for *P. glacialis* across different periods.

The tool “Distribution Changes Between Binary SDMs” was applied to compare habitat suitability changes, which were mainly quantified by comparing the areas of current and future suitable habitats. Based on these changes, habitat dynamics were categorized as expansion (gain of suitable area), contraction (loss of suitable area), or stability (no substantial change).

To quantify the spatial shifts of suitable habitats across different periods, we extracted habitat centroids for each time slice using the “Centroid Changes (Lines)” tool. This tool will calculate the distributional changes between two binary SDMs (e.g., current and future SDMs) and will output a vector file depicting the magnitude and direction of change (based on centers of the species ranges‐‐ their centroids). The centroid is calculated by averaging the latitude and longitude of all suitable/presence input pixels. The detailed processing workflow is described as follows: First, basic centroid datasets were generated via the “Centroid Changes (Lines)” tool. Second, coordinate values of each centroid were extracted with the Add Field tool. Third, all centroid datasets were reprojected to a projected coordinate system through Data Management geoprocessing tools to eliminate calculation errors in migration distance caused by geographic coordinate systems. Finally, spatial analysis tools were utilized to compute the migration distances between centroids of multiple periods based on the reprojected centroid vector data. Predicted centroid migration vectors and distances were mapped to visualize habitat shifts over time (Brown 2014). The shifting rate was determined by calculating the centroid shifting lengths and time intervals (Ran et al. 2024). To evaluate elevational changes associated with centroid shifts, we extracted elevation values from the WorldClim v2.1 elevation layer at the centroid coordinates of current and future suitable habitats using the ‘Extract Multi Values to Points’ tool in ArcGIS 10.4.

## 3 Results

### 3.1 Model Validation

The results from the 10 repeated random sub‐sampling (75:25 holdout) runs for all 12 modeling methods demonstrated strong predictive performance, with average AUC values ≥ 0.80 and TSS ≥ 0.61, indicating high model reliability across different variable combinations (Figures 1, S5 and S6). Both variable combinations indicate that the model performance is excellent. To enhance prediction accuracy, only models with AUC ≥ 0.95 and TSS ≥ 0.85 were selected for constructing the final ensemble models. For the climate‐only variable combination, the selected models were Generalized Additive Model (GAM), Random Forest (RF), and Maximum Entropy (MaxEnt). For the full‐variable combination (climate, elevation, HPD, and NDVI), the chosen models were Multivariate Adaptive Regression Splines (MARS), RF, and MaxEnt (Figure S7). These optimized selections ensured robust and high‐precision predictions of *P. glacialis* habitat distribution. The Boyce index reached 0.963 for the climate‐only variable subset (Figure S8a) and increased to 0.983 after incorporating supplementary environmental predictors (Figure S8b). The Sørensen and Jaccard similarity coefficients (Table S6) further quantified the spatial overlap of suitable habitats between current and future projections, indicating moderate consistency in the spatial patterns of predicted distributions.

**FIGURE 1:**
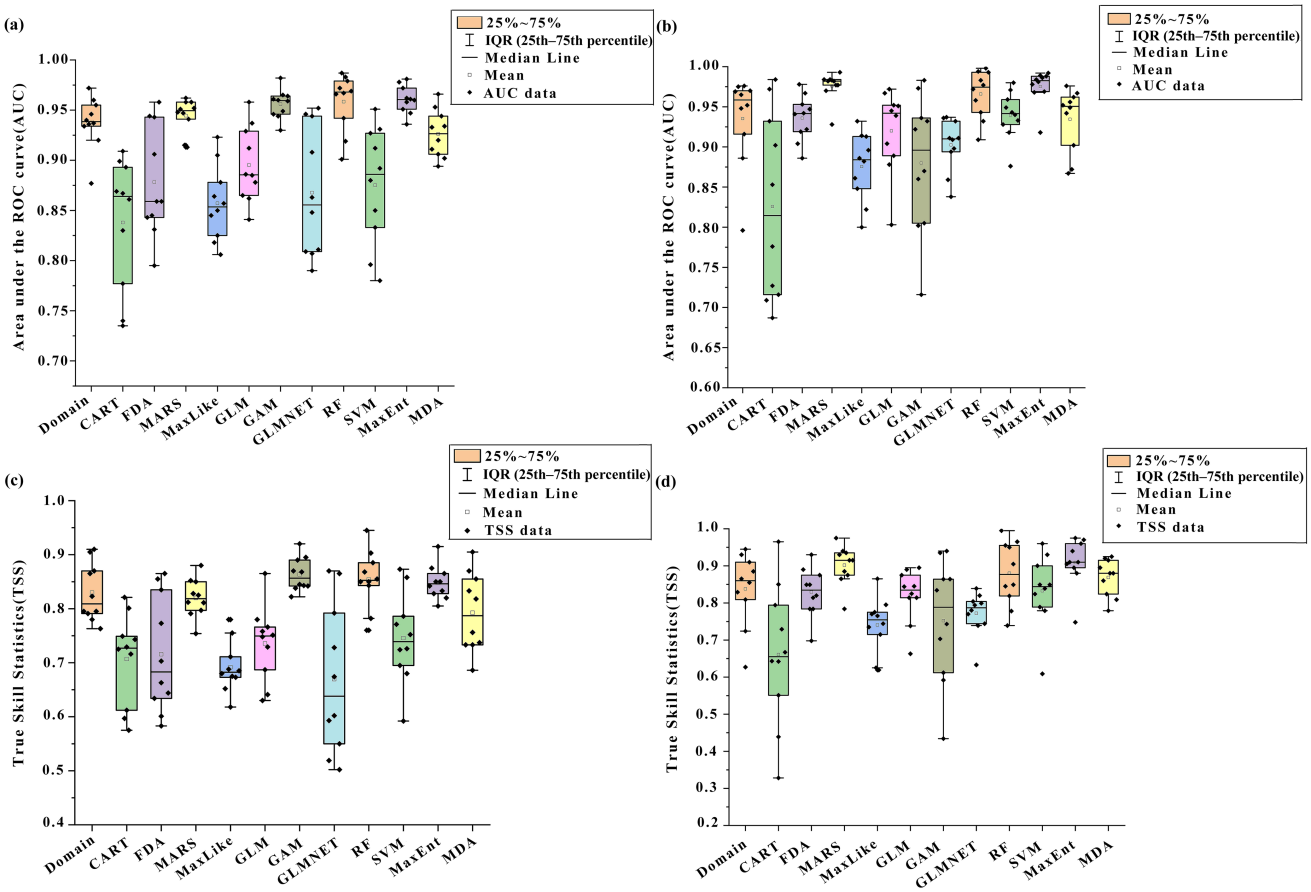
The area under the receiver operating characteristic curve (AUC) and true skill statistics (TSS) values of 12 commonly used species distribution models under different combinations of variables. (a) AUC values based on the climate‐only model; (b) AUC values based on the full‐variable model (climate, elevation, HPD, and NDVI); (c) TSS values based on the climate‐only model; (d) TSS values based on the full‐variable model (climate, elevation, HPD, and NDVI).

### 3.2 Environmental Variables

The importance values reported in Figure 2 were derived from the ‘getVarImp’ function across the selected algorithms for each variable combination. For the climate‐only model, the combined importance reflects the averaged normalized importance from GAM, RF, and MaxEnt; for the full‐variable model, it reflects the averaged normalized importance from MARS, RF, and MaxEnt. The original importance values from each run were normalized to a 0–100 scale prior to averaging, as described in the Methods. Under the climate‐only variable combination (Figure 2a), temperature seasonality (BIO4) had the highest contribution to the predicted distribution of *P. glacialis* (44.12%), followed by precipitation of the warmest quarter (BIO18, 32.96%), precipitation seasonality (BIO15, 10.3%), and mean diurnal range (BIO2, 4.95%). Mean temperature of the wettest quarter (BIO8) contributed the least (0.89%). When HPD, altitude, and NDVI were incorporated into the model (Figure 2b), BIO4 remained the most influential variable (39.26%), followed by BIO2 (21.12%) and BIO18 (19.97%), while NDVI had the least impact (0.07%).

**FIGURE 2:**
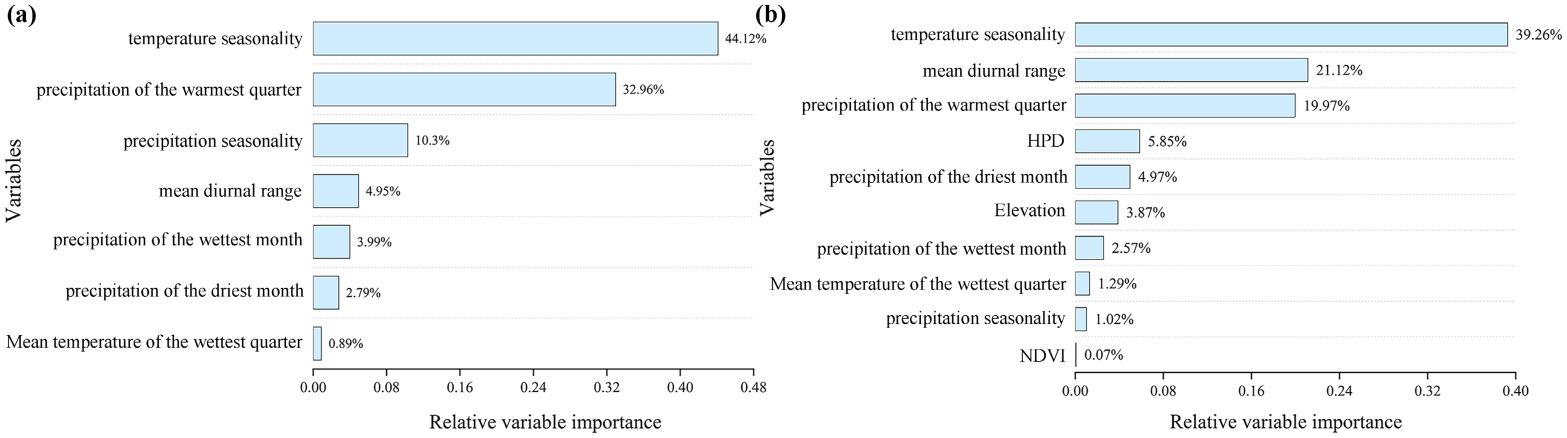
Variable importance for predicting habitat suitability of *P. glacialis* using different combinations of environmental variables. (a) Based on the climate‐only model; (b) Based on the full‐variable model (climate, elevation, HPD, and NDVI).

Despite slight variations in variable rankings across model combinations, temperature‐ and precipitation‐related factors consistently emerged as the primary determinants of the species' ecological niche and habitat suitability. Among these, temperature played the most significant role, underscoring its importance in shaping *P. glacialis* distribution. The response curves illustrate how the probability of occurrence varies in relation to environmental variables (Figures 3 and S9). As shown in Figure 3, the species favors habitats where BIO2 ranges from 7.97°C to 8.22°C, with an optimum at 8.09°C. The response curve for BIO4 followed a unimodal pattern, peaking at 904.39. When BIO8 exceeded 6.08°C, the probability of occurrence increased gradually. Conversely, when precipitation of the wettest month (BIO13) exceeded 512.12 mm, the occurrence probability declined slightly. A similar pattern was observed for precipitation of the driest month (BIO14), where values above 3.36 mm led to a gradual decline, while values below this threshold caused a sharp drop. Furthermore, the probability of occurrence decreased when BIO15 exceeded 22.67 mm, while values of BIO18 above 10 mm were associated with increased occurrence probability.

**FIGURE 3:**
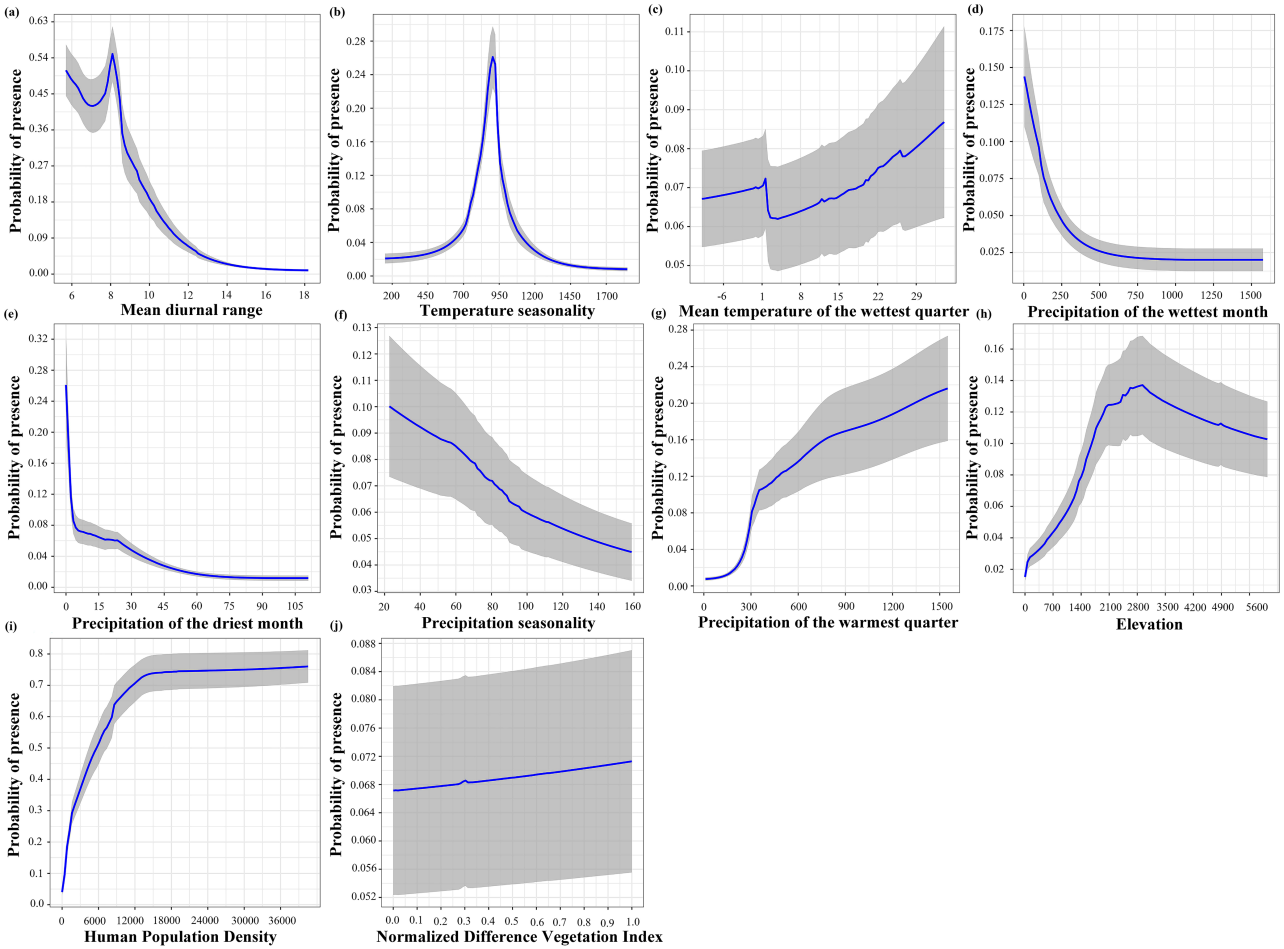
Response curves for ten environmental variables, showing the predicted species presence probability when each variable is used independently for modeling. The blue lines represent the average response across 10 replicate ensemble model runs; gray bands denote mean ± one standard deviation. (a) Mean Diurnal Range (Mean of monthly (max temp—min temp)) (BIO2); (b) Temperature seasonality (standard deviation ×100) (BIO4); (c) Mean temperature of wettest quarter (BIO8); (d) Precipitation of wettest month (BIO13); (e) Precipitation of driest month (BIO14); (f) Precipitation seasonality (Coefficient of variation) (BIO15); (g) Precipitation of warmest quarter (BIO18); (h) Elevation; (I) Human population density (HPD); (j) Normalized difference vegetation index (NDVI).

Among non‐climatic factors, the highest probability of occurrence was observed at an altitude of 2931.42 m. When HPD exceeded 12,690 people/km^2^, the occurrence probability stabilized. For NDVI, a gradual increase in occurrence probability was observed as values ranged from 0 to 1. Overall, the response curve trends in Figure 3 were largely consistent with those shown in Figure S9, with minor discrepancies noted for BIO8, BIO13, and BIO18.

### 3.3 The Current and Future Potential Distribution of *P. glacialis*

The spatial uncertainty across the three GCMs is shown in Figure S10. Notably, low uncertainty (yellow areas) was predominantly observed across the Northwest and Tibetan Plateau, indicating high consensus among models. In contrast, high uncertainty (orange/red areas) was concentrated in the complex terrain of central‐eastern China (e.g., Sichuan, Hubei, and Henan), indicating significant discrepancies among GCMs in these regions.

Different variable combinations were used to estimate the current suitable habitat range of *P. glacialis* (Figure 4 and Table S7). The model based solely on climate variables predicted a larger suitable area compared to the model incorporating altitude, HPD, and NDVI. Nonetheless, the spatial distribution of highly suitable habitats remained largely consistent across models, with core regions concentrated in the provinces of Gansu, Sichuan, Shanxi, Chongqing, Hubei, Henan, Shaanxi, Anhui, Jiangsu, Zhejiang, and Shandong. It should be noted that all comparisons between current and future distributions presented in Figures 5, 6, 7 are based exclusively on the climate‐only model. The full‐variable model (including climate, elevation, HPD, and NDVI) was used only for the current potential distribution shown in Figure 4b as a supplementary representation incorporating anthropogenic and vegetation constraints and was not involved in any temporal comparison. This ensures that the observed shifts in suitable habitat area and centroid displacement reflect climate‐driven changes rather than differences in predictor composition.

Under future climate scenarios (Figure 5 and Table S8), a slight reduction in suitable habitat is projected. Low‐suitability regions increased significantly, moderately suitable regions decreased substantially, and highly suitable regions disappeared entirely (Figure 5 and Table S8). The rate of habitat loss from the present to future periods was similar under both SSP1‐2.6 and SSP5‐8.5 scenarios. In the 2030s and 2050s, the SSP1‐2.6 scenario predicted slightly greater suitable area than SSP5‐8.5. However, by the 2070s, the SSP5‐8.5 scenario projected a modest expansion, suggesting that *P. glacialis* may retain some survival potential under high greenhouse gas emission conditions in the long term.

**FIGURE 4:**
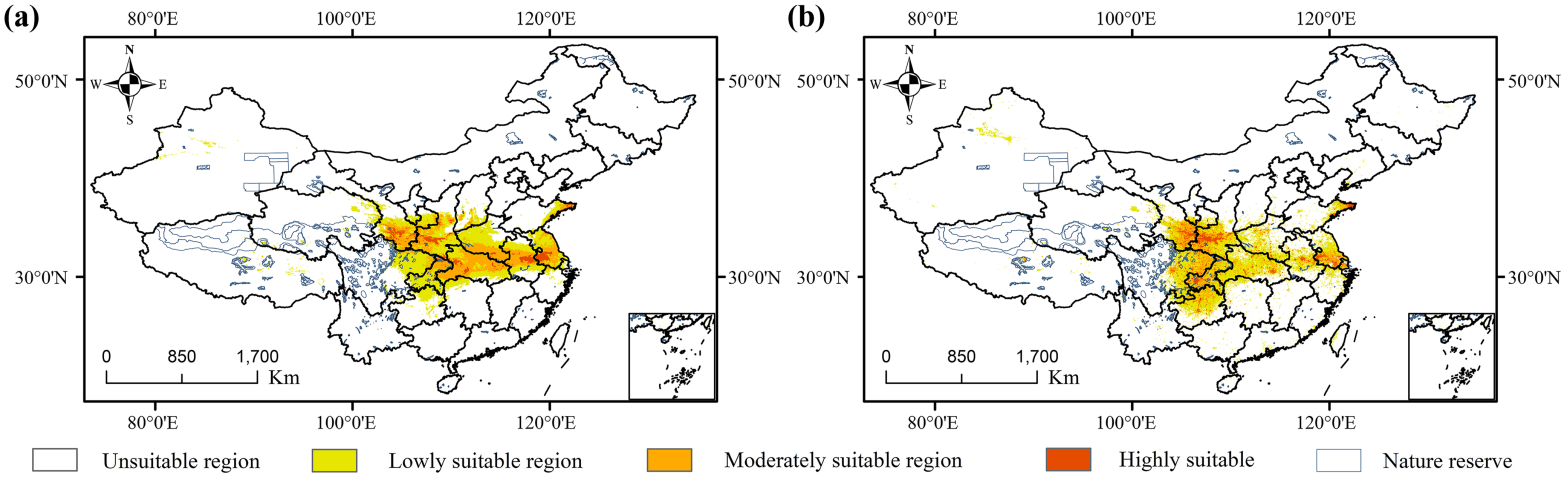
Current potential habitat distribution of *P. glacialis* . (a) Based on the climate‐only model; (b) Based on the full‐variable model (climate, elevation, HPD, and NDVI), shown as a supplementary representation of contemporary habitat constraints and not used for temporal comparisons with future projections.

**FIGURE 5:**
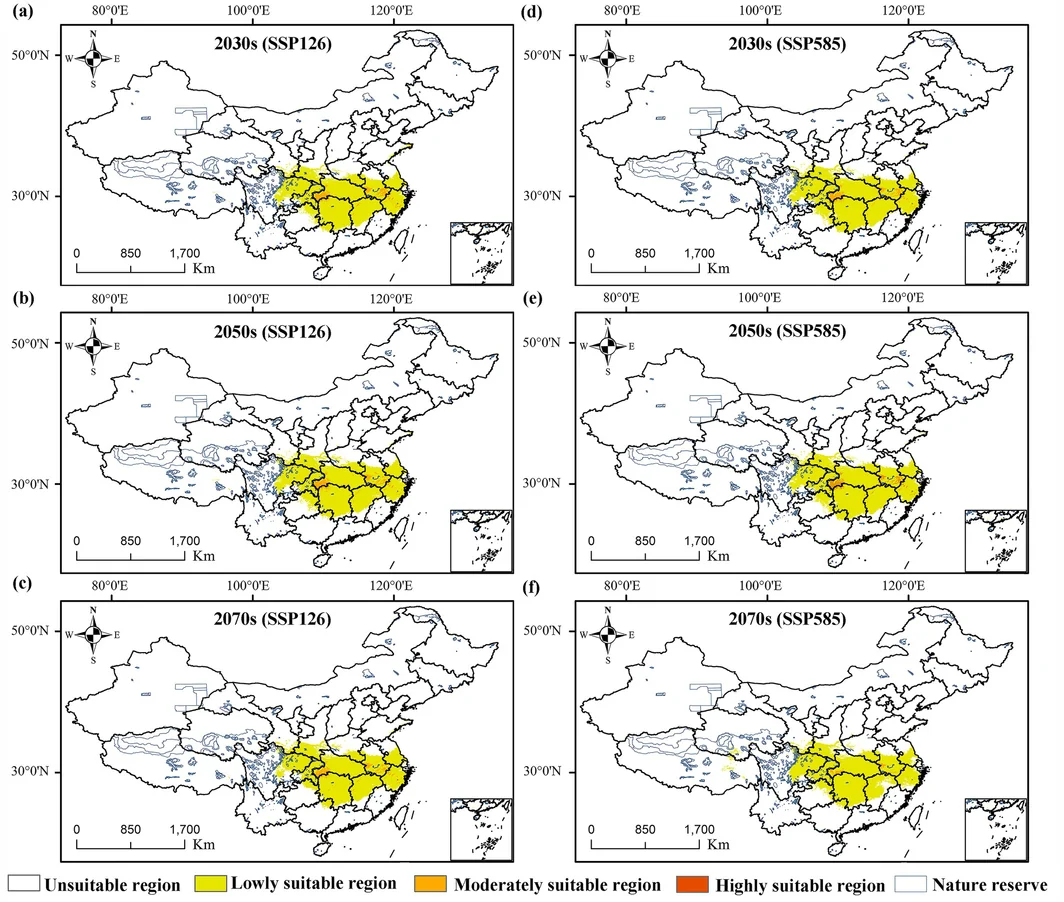
Future potential habitat distribution of *P. glacialis* under SSP scenarios and time periods, projected by the climate‐only model.

Overall, a southward shift in the species' habitat is expected. The extensive range of low‐suitability areas indicates that the species faces a genuine extinction risk. Meanwhile, most of China's National Nature Reserves are located in the northwest and southwest regions, creating a potential mismatch between conservation priorities and future habitat suitability. This highlights the need for adaptive conservation strategies to safeguard the long‐term survival of *P. glacialis* under changing climate conditions.

### 3.4 Habitat Suitability Area and Dynamic Changes Under Different Climate Scenarios

Relative to current climate conditions, the patterns of habitat expansion and contraction under all future scenarios were broadly similar (Figure 6 and Table S9). These comparisons are based on the climate‐only model. In the 2030s and 2050s, both SSP1‐2.6 and SSP5‐8.5 projected slightly more expansion of suitable habitat than contraction. However, by the 2070s, this trend reversed, with habitat loss surpassing habitat gain. Future habitat expansion is expected to occur mainly in Hunan, Jiangxi, and Zhejiang provinces, while significant habitat contraction is predicted in Gansu, Shaanxi, and Henan. Meanwhile, suitable habitats in western Sichuan, Hubei, southern Shaanxi, Chongqing, and central Jiangsu are expected to remain relatively stable.

**FIGURE 6:**
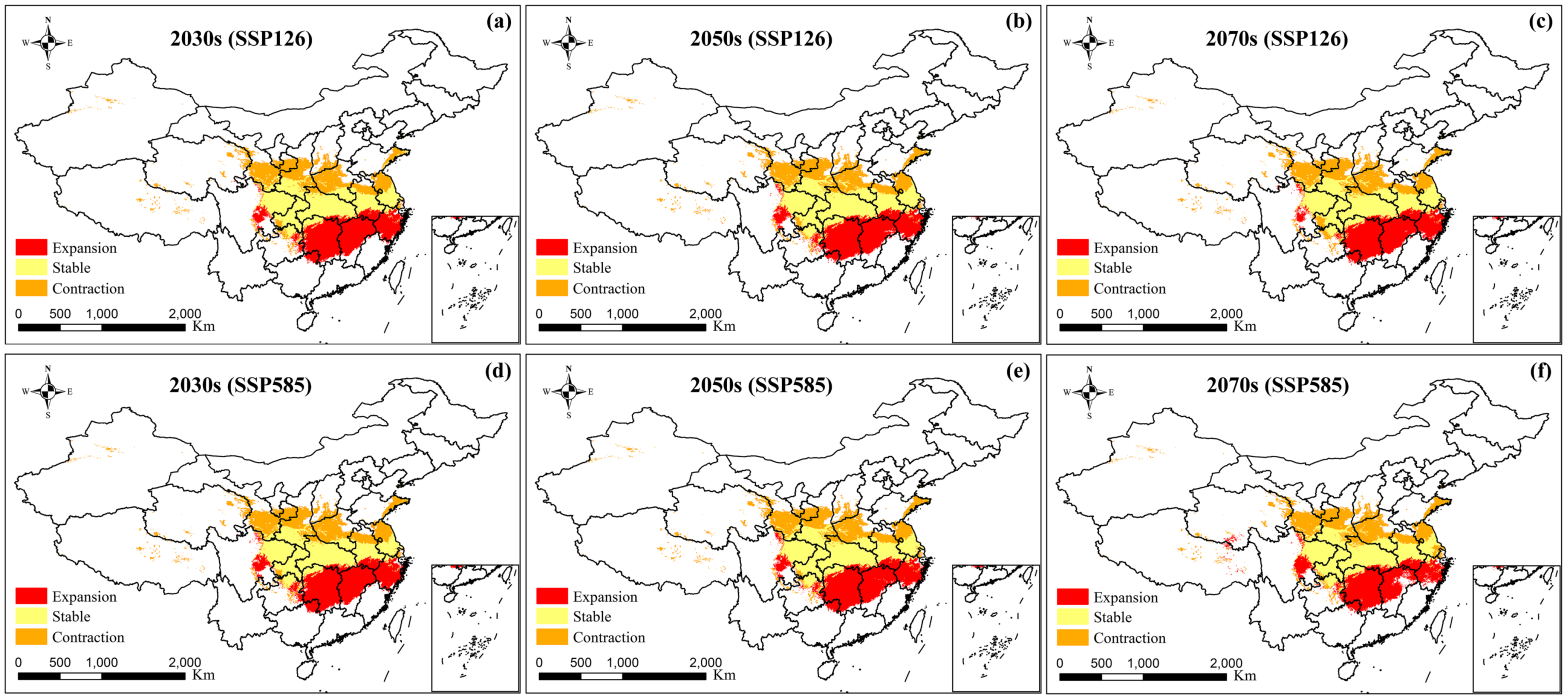
Changes in the potential habitat distribution of *P. glacialis* under future climate scenarios based on SSPs, derived from the climate‐only model.

At present, the geographic centroid of *P. glacialis* suitable habitats is located in Hubei Province (110.57° E, 32.04° N) (Figure 7). Under the SSP1‐2.6 scenario, the centroid is projected to shift southeastward by approximately 356 km in the 2030s, after which it will remain stable through the 2050s and 2070s. A similar pattern is projected under SSP5‐8.5, with a southeastward shift of 357 km by the 2030s, followed by stabilization in subsequent periods (Figure 7 and Table S10). These findings suggest that, while some habitat displacement is likely, the overall geographic shift of *P. glacialis* 's suitable habitat will be relatively limited under future climate scenarios.

To assess whether the observed southeastward centroid shifts correspond to changes in elevation, we extracted elevation values (from the WorldClim v2.1 elevation layer) at each centroid location for the current and future periods. The current centroid elevation is 354 m. Under the SSP1‐2.6 scenario, the centroid elevation decreased to 27 m in the 2030s, 30 m in the 2050s, and 27 m in the 2070s, representing a decrease of 324–327 m relative to the current centroid elevation (Table S11). Under SSP5‐8.5, the centroid elevation decreased to 30 m, 31 m, and 36 m in the 2030s, 2050s, and 2070s, respectively, representing a decrease of 318–324 m. These results confirm that the southeastward range shift is accompanied by a consistent and substantial downward shift in elevation (from ~350 to ~30 m), supporting the inference of low‐altitude migration. This finding is consistent with the known elevational range of *P. glacialis* (Zhao et al. 2023).

**FIGURE 7:**
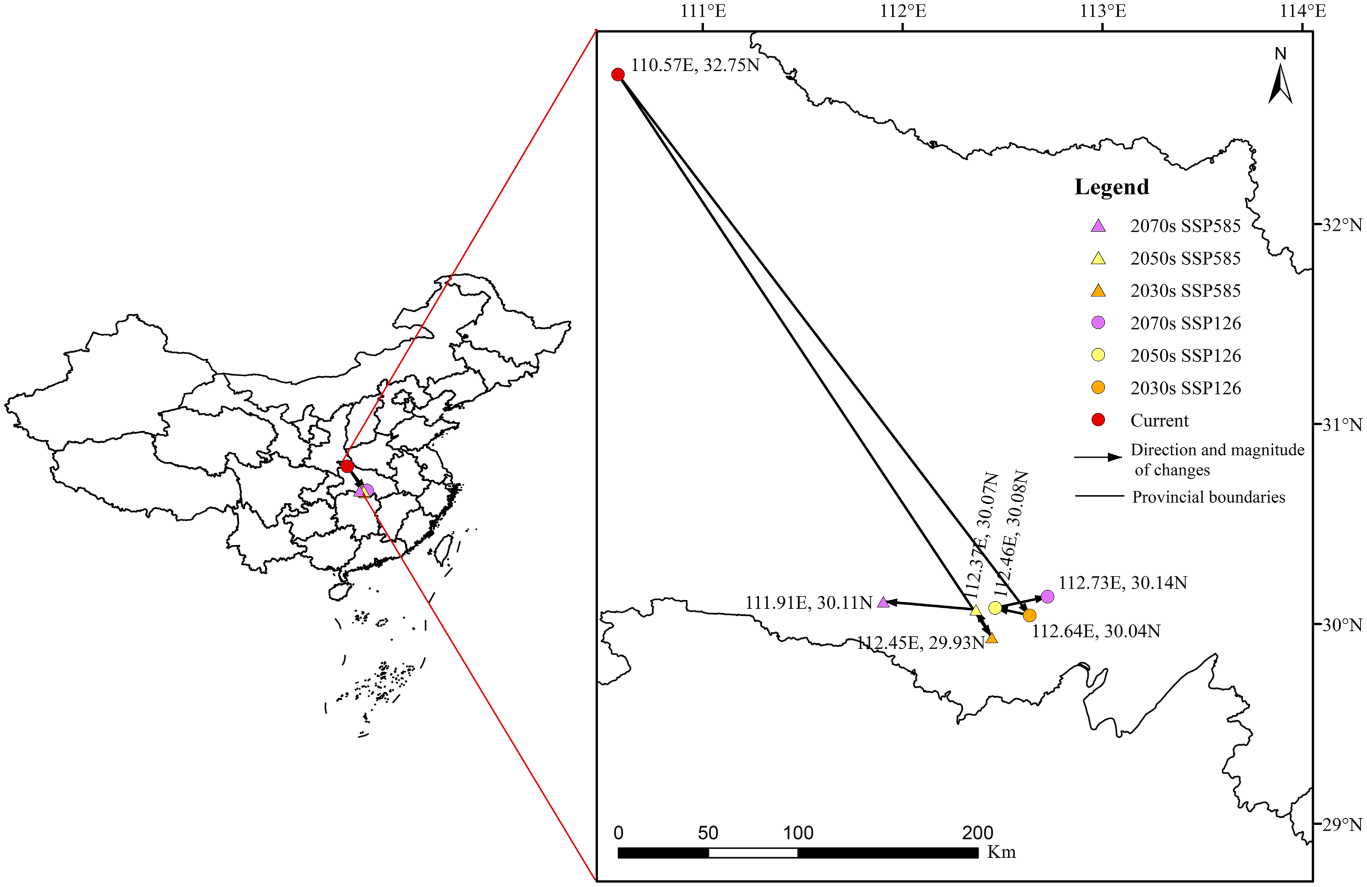
Shifts in the core distribution of *P. glacialis* under different future climate scenarios, derived from the climate‐only model.

## 4 Discussion

The genus *Parnassius* is renowned for its rare species, most of which inhabit high‐altitude plateau regions (Zhou 1999). Among them, the Glacial Apollo *P. glacialis* is a protected butterfly species and the only representative of the genus found in Jiangnan, China (Zhou 1999). This study is the first to apply an ensemble species distribution model (SDM) to predict the spatial and temporal distribution of *P. glacialis* in China from a biogeographical perspective. Multiple quantitative evaluation metrics confirmed that the ensemble models constructed based on two sets of environmental variables achieved robust and stable predictive performance, enabling reliable delimitation of current suitable habitats and credible projections of future distributional shifts under diverse climate change scenarios. The climate‐only model was used for all temporal comparisons (Figures 5, 6, 7), whereas the full‐variable model (including HPD and NDVI) served only to characterize current habitat constraints (Figure 4b) and was not used for future projections.

The high standard deviations observed in central‐eastern China (Figure S10) likely result from the intricate topographical and monsoon interactions in these regions, which different GCMs simulate with varying degrees of accuracy. Therefore, conservation planning and habitat management strategies in these specific high‐uncertainty zones should be interpreted with caution and implemented adaptively.

Previous applications of ensemble models have shown that the six single models, namely MaxEnt, GLM, GAM, GBM, MARS, and RF, exhibit high stability and satisfactory predictive accuracy, making them suitable for studies on many species. Among them, MaxEnt and RF are the most widely used (Chen, Longzhu, et al. 2023; Yu et al. 2023; Ran et al. 2024; Yang, Gu, and Wang 2024; Yang, Huo, et al. 2024; Liu et al. 2025). As can be seen from our results (Figure 1), although differences exist among individual models, the evaluation metrics AUC and TSS are positively correlated, consistent with previous findings.

The predictive performance of MaxEnt is slightly better than that of RF. This finding do not agree with the statement of Ran et al. (2024). Previous studies have demonstrated that species distribution models (SDMs) exhibit distinct applicable conditions, and the intrinsic geographic distribution characteristics of target species act as core limiting factors governing model performance (Fei et al. 2025). The geographic range, spatial aggregation pattern and spatial autocorrelation of occurrence records can substantially distort predictive accuracy. Compared with the MaxEnt, the RF is far more susceptible to such spatial confounding effects and yields less stable outputs. The dataset used in this study has a smaller sample size than that adopted by Ran et al. (2024), representing a typical small‐sample scenario (Ran et al. 2024). Comparative evaluations of multiple modeling algorithms have proven that MaxEnt delivers superior robustness when trained on limited occurrence data (Li and Wang 2013), which is further validated by our comprehensive model evaluation metrics.

Insect species exhibit divergent habitat responses under ongoing global warming, reflecting taxon‐specific climatic adaptability. Previous SDM simulations documented a fluctuating “decrease‐then‐increase” habitat trend for Zyginelline insects and a continuous habitat contraction trend for *Papilio xuthus* under identical future climate projections (Ran et al. 2024; Liu et al. 2025). Analogously, our projections demonstrated an overall contraction of suitable habitats for *P. glacialis* under both low‐ and high‐emission scenarios (Figures 4 and 5), further confirming that climate change poses pervasive habitat loss risks for vulnerable montane insect species.

Discrepancies exist in the predictive outputs of various models, which stem from intrinsic model properties and external input conditions. Intrinsic factors: First, algorithms fundamentally differ in mechanism and fitting capacity. For instance, MaxEnt relies on maximum entropy theory and focuses on characterizing the probability distribution between species presence records and background points, whereas Random Forest (RF) is a machine learning algorithm proficient in capturing complex nonlinear interactions (Phillips et al. 2006; Breiman 2001). Second, studies adopt distinct tuning strategies for model hyperparameters. External factors: On one hand, models vary in their capacity to detect nonlinear relationships among environmental predictors, resulting in species‐specific fitting performance (Ran et al. 2024; Liu et al. 2025). On the other hand, incomplete occurrence datasets will exacerbate predictive divergence across models. Furthermore, whether ecological processes are incorporated and the variation of ecological constraints embedded within model frameworks will jointly shape the final projections (Beaumont et al. 2008).

Therefore, the selection of algorithms should comprehensively consider the biological traits of target species, environmental variables, and the applicable scope of each model. In addition, future relevant research can implement systematic grid search for hyperparameter tuning to guarantee robust model performance.

Beyond algorithm selection, the sampling strategy for pseudo‐absence and background points also warrants methodological attention. In this study, we adopted a random sampling strategy without applying exclusion buffers around occurrence records. This decision was primarily based on three considerations. First, the calibration area (MCP with a 50‐km buffer) is relatively constrained in extent; imposing exclusion buffers could artificially remove environmentally similar background pixels adjacent to occurrence points, potentially leading to conservative estimates of niche boundaries. Second, the prior application of spThin with a 10‐km thinning distance had already reduced spatial autocorrelation among occurrence records, thereby mitigating the concern that pseudo‐absence or background points might fall too close to presence points. Nevertheless, we acknowledge that this approach does not account for potential sampling biases inherent in the original occurrence data, nor does it address the possibility that unsampled environmentally suitable sites may be misclassified as pseudo‐absences. Future studies with larger occurrence datasets should consider incorporating target‐group background sampling or environmentally stratified pseudo‐absence selection to further improve model discrimination.

Our ensemble model predictions indicated that the species' predicted habitat was primarily concentrated in central‐eastern China—covering provinces such as Gansu, Sichuan, Shanxi, Chongqing, Hubei, Henan, Anhui, Jiangsu, Zhejiang, and Shandong—which closely matches its known distribution (Zhou 1999; Wang et al. 2019; Hu et al. 2022). These results indicate that the ensemble models effectively capture the species' current range and exhibit strong predictive accuracy and reliability.

An assessment of China's national nature reserves reveals that these protected areas are primarily concentrated in the northwest and southwest. However, many regions offering medium‐to‐high suitability for *P. glacialis* currently lack dedicated conservation initiatives. As a rare plateau species of both ecological and scientific value, *P. glacialis* is listed in China's species protection regulations, highlighting the urgency of optimizing regional conservation planning for this vulnerable species.

Simulations under diverse future climate scenarios reveal that the suitable distribution range of *P. glacialis* —the sole alpine Apollo butterfly capable of occupying low‐elevation habitats—will shift toward low‐altitude areas in southeastern Hubei Province (Figure 7), accompanied by a drastic reduction in the area of moderately and highly suitable habitats (Figure 5). Temperature and precipitation are the primary climatic drivers identified by our model, and they are well known to jointly regulate insect growth and development through both direct physiological effects and indirect effects on host plant resources (Ran et al. 2024). Although host plant distributions were not directly included as model predictors, it is reasonable to infer that climate‐driven changes in host plant availability could further affect the persistence of *P. glacialis* populations. Similarly, while phenological synchrony between insects and their host plants was not explicitly modeled, global warming is widely documented to disrupt such synchrony (Ran et al. 2024), representing a potential additional threat that warrants future investigation. Phenological synchronization between herbivorous insects and their host flora is an essential prerequisite for the insects' normal growth and reproduction. Previous research has verified that plant anti‐herbivore defensive capacity intensifies with elevation (Rasmann et al. 2014). Although plant defensive traits were not modeled explicitly, this elevational gradient in plant defense—combined with the projected downward shift of *P. glacialis* (Table S11)—may expose populations to different herbivory pressures in their future low‐elevation habitats, though this remains a hypothesis to be tested. Similarly, while our model did not explicitly incorporate microclimatic refugia, it has been suggested that species facing climate‐induced habitat loss may seek thermally stable microhabitats (McCain and Garfinkel 2021); whether *P. glacialis* will successfully find such refugia in its future low‐elevation range requires further field‐based investigation.

The southeastward and downward shift of *P. glacialis* projected across multiple SSPs—with centroid elevation decreasing by approximately 320 m from its current elevation of 354 m to ~30 m under both SSP1‐2.6 and SSP5‐8.5 scenarios (Table S11), consistent with the species' capacity to disperse into low‐altitude environments (Zhao et al. 2023)—represents a typical climate adaptation response of the species to track favorable living conditions. Analogous biogeographic range displacements have been documented across various taxonomic groups. Historical distribution simulations for two extinct British butterfly species, *Aporia crataegi* and *Polyommatus semiargus*, also predicted their populations would retreat southeastward within the United Kingdom (Carroll et al. 2009). Threatened amphibian populations are likewise expected to migrate toward lower latitudes to cope with rising temperatures (He et al. 2024). Existing climate modeling demonstrates prominent spatial heterogeneity in warming magnitudes across China: warming rates in Northwest China, Northeast China and the Qinghai–Xizang Plateau will exceed the national average, while annual precipitation volume and intensity will increase markedly in southeastern China (Feng et al. 2011). The geographic ranges of butterflies are tightly constrained by the spatial distribution of their larval host plants (Filazzola et al. 2020). Further projections for the host plants of *P. glacialis* larvae indicate that suitable habitats for host flora will span the southern subtropical, northern subtropical, southern temperate and mid‐temperate zones in the future (Chen, Longzhu, et al. 2023). Accordingly, considering the unique ecological requirements of this butterfly, *P. glacialis* will disperse to regions with milder warming to secure habitats with stable thermal, hydric and ecosystem conditions, ultimately forming a pattern of southeastward downhill migration as an adaptive strategy against global warming. Nevertheless, such low‐elevation refugia impair the overall ecological fitness of *P. glacialis* and reduce habitat suitability, thereby elevating its long‐term risk of extinction. To alleviate the survival pressures imposed by multiple stressors, this study proposes establishing new protected areas across North, Central and East China, with priority given to in situ conservation measures to sustain viable wild populations in the long run.

A comprehensive understanding of the effects of human activities and climate change on the distribution of *P. glacialis* in China is essential for developing and implementing effective conservation measures and promoting the recovery and return of populations. Human population density is positively correlated with species extinction risk. Human activities cause habitat fragmentation and ecological degradation, thereby reducing habitat suitability and impairing the survival and reproductive capacity of species (Luck 2007). There is a significant interaction between climatic drivers and human population density. Climatic drivers amplify the negative impacts of human activities, while human disturbances restrict the migration and adaptive capacity of species in response to climatic drivers (Alipour and Walas 2023). In our ensemble models, temperature, precipitation, and elevation consistently emerged as the primary predictors of habitat suitability, with temperature seasonality (BIO4) contributing the most (39.26%). This underscores temperature as the dominant factor influencing *P. glacialis* distribution. While most environmental variables showed similar response trends across different model combinations, some discrepancies were observed in the responses of mean temperature of the wettest quarter (BIO8), precipitation of the wettest month (BIO13), and precipitation of the warmest quarter (BIO18). These differences likely result from algorithmic variability among models (Marmion et al. 2009; Duncan et al. 2021).

Our findings align with prior studies on butterfly distributions, which similarly identify temperature, precipitation, and altitude as key drivers of species ranges (Ashton et al. 2016), further validating the robustness of our ensemble model. Moreover, the occurrence probability of *P. glacialis* increases with higher NDVI values, which is consistent with the findings of Habel et al. (2022) in their study on *Euphydryas maturna* (Habel et al. 2022). The high spatial congruence between environmental response curves and raw occurrence data distributions (Figures 3 and S9; Tables S12–S21) provides robust indirect evidence for the high precision and credibility of our model outputs.

Under progressive global warming, most terrestrial species redistribute their ranges toward climatically favorable regions to maintain population viability (Macfadyen et al. 2018). Both SSP1‐2.6 and SSP5‐8.5 future scenarios projected continuous suitable habitat loss for *P. glacialis* , which is consistent with the climate‐driven range contraction of alpine *Parnassius* species in the Hengduan Mountains (Yu et al. 2023). Our projections suggest that although the overall suitable habitat for *P. glacialis* may remain relatively stable, noticeable shifts in its distribution are likely between present‐day and future climate scenarios. This discrepancy is likely attributable to variable climate conditions, which can drive either expansion or contraction of species' suitable habitats (Liu et al. 2019, 2020; Santana Jr et al. 2019; Aidoo et al. 2023).

During the 2030s and 2050s, habitat expansion is predicted under the SSP1‐2.6 due to more favorable conditions. However, under the high‐emissions SSP5‐8.5 scenario, a notable contraction in suitable habitat is expected, while it is projected to be more pronounced in Qinghai and Tibet compared to SSP1‐2.6. As a global biodiversity hotspot, the Qinghai–Tibet Plateau supports a high concentration of endemic and endangered species (Sun et al. 2021). Forecasted increases in temperature and humidity in this region (Nan et al. 2005; Li et al. 2010) may explain its growing suitability for *P. glacialis* . These results underscore the need to incorporate climate change projections into conservation strategies to ensure the long‐term survival of *P. glacialis* and other similarly vulnerable species.

We acknowledge several methodological limitations regarding hyperparameter configuration in this study. First, all algorithms were run with default parameters from the ‘sdm’ package without systematic tuning. While this approach reduces overfitting risks under small sample conditions (*n* = 45), it may not achieve optimal performance for each individual algorithm. Second, the lack of independent tuning datasets prevented us from evaluating the sensitivity of model outputs to alternative hyperparameter settings. Third, although the ensemble framework mitigates some uncertainty associated with individual algorithm configurations, future studies with larger occurrence datasets should incorporate systematic tuning strategies (e.g., grid search with cross‐validation) to further enhance model robustness and predictive accuracy.

Our 10 repeated random sub‐sampling (75:25 holdout) runs employed random data partitioning rather than spatially explicit blocking. Because *P. glacialis* occurrence records exhibit spatial clustering, this approach may lead to optimistic performance estimates—a well‐documented issue in species distribution modeling (Roberts et al. 2017; Valavi et al. 2022). To partially mitigate this concern, we applied spThin with a 10‐km thinning distance prior to modeling, which reduced the most severe spatial autocorrelation among occurrence points. However, we did not perform spatially blocked cross‐validation (e.g., spatial block CV), as the limited sample size (*n* = 45) would have resulted in excessively small training sets and unstable model fits. We therefore caution that the reported AUC and TSS values should be interpreted with this caveat in mind, and we encourage future studies with larger occurrence datasets to adopt spatially explicit validation strategies for more conservative performance assessments.

We further acknowledge that our use of non‐climatic variables (HPD and NDVI) in the current full‐variable model precludes direct comparison of habitat area changes between that model and future projections, which rely solely on climatic predictors. However, as explicitly stated in the Methods and Results, all temporal comparisons of habitat dynamics (Figures 5, 6, 7) were based exclusively on climate‐only models, ensuring that the observed shifts reflect climate‐driven changes rather than artifacts of changing predictor composition. The full‐variable model was constructed solely as a supplementary characterization of current habitat constraints for conservation planning purposes and was not used in any temporal comparison. We caution that the current distribution shown in Figure 4b should be interpreted in this context and not as a baseline for future comparisons.

We acknowledge that several ecological processes discussed—including host plant distribution shifts, phenological synchrony, plant defensive traits, and microclimatic refugia—were not explicitly modeled in this study. These factors represent important avenues for future research. Integrating host plant distribution models, phenological data, and high‐resolution microclimate layers into SDM frameworks would enable more direct testing of the hypotheses raised here and improve the mechanistic understanding of *P. glacialis* responses to climate change.

## 5 Conclusion

This study employed an ensemble species distribution model to evaluate current and projected habitat suitability of the Glacial Apollo butterfly, *P. glacialis* . The results highlight temperature—particularly temperature seasonality—as the key factor influencing its spatial distribution. Under future climate scenarios, a notable reduction in suitable habitats is anticipated, increasing the species' risk of extinction. To counter this threat, the study emphasizes the urgent need for in situ conservation measures, informed policy action, and ongoing habitat monitoring, coupled with experimental research into the species' climate resilience. Critically, it advocates for the establishment of new conservation zones in North, Central, and East China to preserve *P. glacialis* populations and sustain its ecological role. These insights provide essential guidance for developing effective conservation strategies in the face of accelerating climate change.

## Author Contributions

**Yongli Wang:** data curation (equal), investigation (equal), methodology (equal), visualization (equal), writing – original draft (equal). **Mingsheng Yang:** methodology (equal), writing – review and editing (equal). **Yupei Hu:** data curation (equal), investigation (equal). **Raul Narciso C. Guedes:** writing – review and editing (equal). **Youssef Dewer:** methodology (equal), writing – review and editing (equal). **Suqin Shang:** conceptualization (equal), funding acquisition (equal), supervision (equal), writing – review and editing (equal). **Xiushan Li:** conceptualization (equal), supervision (equal).

## Funding

This study was supported by Investigation on the Resources of Butterflies in Liancheng National Nature Reserve, Gansu Province and the population distribution model of their dominant species (GSAU‐JSFW‐2025‐15), the Ministry of Science and Technology of the People's Republic of China (Grant No. 2022YFE0115200), and Gansu Agricultural University (GSAU‐JSFW‐2022‐082).

## Conflicts of Interest

The authors declare no conflicts of interest.

## Supporting information


**Figure S1:** Distribution records of the *Parnassius glacialis*. (a) *Parnassius glacialis*; (b) Final distribution points used for modeling.
**Figure S2:** The Pearson's correlation analyses of 19 bioclimatic variables. (a) Correlation matrix of the original 19 bioclimatic variables; (b) Correlation matrix of the standardized 19 bioclimatic variables.
**Figure S3:** Pearson correlation analysis among the environmental variables. (a) Correlation matrix of the 22 candidate environmental variables (19 bioclimatic variables, elevation, HPD, and NDVI); (b) Pearson correlation between Human Population Density (HPD) and Human Footprint Index (HFP).
**Figure S4:** The final Calibration Area (CA, semi‐transparent polygon) for *P. glacialis* in China, derived from the Minimum Convex Polygon (MCP, green dashed line) of 45 validated occurrence records (purple dots) expanded by a 50‐km dispersal buffer. The inset map shows the full national boundaries including the South China Sea islands. The main map is zoomed‐in on central‐eastern China with a semi‐transparent topographical background (Hillshade) to show how the buffer follows mountain ridgelines while avoiding deep valleys.
**Figure S5:** The receiver operating characteristic (ROC) curves of the 12 ecological niche models under the climate‐only model.
**Figure S6:** The receiver operating characteristic (ROC) curves of the 12 ecological niche models under the full‐variable combination (climate, elevation, HPD, and NDVI).
**Figure S7:** Receiver operating characteristic (ROC) curves of the three individual models selected for ensemble modeling under different variable combinations. (a) GAM, (b) RF, and (c) MaxEnt based on the climate‐only model; (d) MARS, (e) RF, and (f) MaxEnt based on the full‐variable model (climate, elevation, HPD, and NDVI).
**Figure S8:** Boyce index under different variable combinations. (a) Based on the climate‐only model; (b) Based on the full‐variable model (climate, elevation, HPD, and NDVI).
**Figure S9:** Response curves for the seven climatic variables included in the climate‐only model, illustrating how the predicted probability of presence changes when the model is created using only that variable. The blue lines represent the average response across 10 replicate ensemble model runs; gray bands denote mean ± one standard deviation. (a) Mean Diurnal Range (Mean of monthly (max temp—min temp)) (BIO2); (b) Temperature seasonality (standard deviation ×100) (BIO4); (c) Mean temperature of wettest quarter (BIO8); (d) Precipitation of wettest month (BIO13); (e) Precipitation of Driest Month (BIO14); (f) Precipitation Seasonality (Coefficient of Variation) (BIO15); (g) Precipitation of warmest quarter (BIO18).
**Figure S10:** Spatial uncertainty (standard deviation, SD) of the predicted suitable habitats for *P. glacialis* across three GCMs (BCC‐CSM2‐MR, IPSL‐CM6A‐LR, and MRI‐ESM2‐0) under two SSP scenarios (SSP1‐2.6 and SSP5‐8.5) and three time periods. (a–c) SD under SSP1‐2.6 for the 2030s, 2050s, and 2070s, respectively; (d–f) SD under SSP5‐8.5 for the 2030s, 2050s, and 2070s, respectively. Higher SD values (orange/red) indicate lower consensus among climate models, while lower SD values (yellow) indicate higher consensus. The inset map shows the South China Sea Islands.
**Table S1:** The occurrence records of *Parnassius glacialis* and their sources.
**Table S2:** The occurrence records used in the ensemble models.
**Table S3:** The environmental variables considered in this study.
**Table S4:** Summary of absence/background types and sampling parameters for each algorithm.
**Table S5:** Summary of default hyperparameter configurations for each algorithm used in the ‘sdm’ package.
**Table S6:** Sørensen and Jaccard Similarity Indices between Current and Future Habitat Suitability.
**Table S7:** The suitable areas of *Parnassius glacialis* under current (×10^4^ km^2^).
**Table S8:** The suitable areas of *Parnassius glacialis* under climate conditions (×10^4^ km^2^).
**Table S9:** Changes of spatial distribution pattern of *Parnassius glacialis* under different climate conditions.
**Table S10:** Centroid shifting distance and speed of *Parnassius glacialis* in different periods.
**Table S11:** Elevation changes of the geographic centroid of *Parnassius glacialis* suitable habitats across different periods.
**Table S12:** BIO2 values at the 45 occurrence points under current climate conditions.
**Table S13:** BIO14 values at the 45 occurrence points under current climate conditions.
**Table S14:** BIO8 values at the 45 occurrence points under current climate conditions.
**Table S15:** BIO13 values at the 45 occurrence points under current climate conditions.
**Table S16:** BIO15 values at the 45 occurrence points under current climate conditions.
**Table S17:** BIO18 values at the 45 occurrence points under current climate conditions.
**Table S18:** BIO4 values at the 45 occurrence points under current climate conditions.
**Table S19:** Elevation values at the 45 occurrence points under current climate conditions.
**Table S20:** NDVI values at the 45 occurrence points under current climate conditions.
**Table S21:** HPD values at the 45 occurrence points under current climate conditions.

## Data Availability

The data that support the findings of this study are openly available in Sciencedb at https://doi.org/10.57760/sciencedb.41395.
