# Supplementary material for: Climate‐Driven Distribution Modeling of the Glacial Apollo Butterfly (Parnassius glacialis) for Biodiversity Conservation
Source: Ecol Evol. 2026 Nov 1;16(11):e74412. doi: 10.1002/ece3.74412 (PMC13724144; doi:10.1002/ece3.74412)
Supplement: Supplementary file 1 [file ECE3-16-e74412-s001.docx]

Supporting Information

Climate-Driven Distribution Modelling of the Glacial Apollo Butterfly (*Parnassius glacialis*) for Biodiversity Conservation

**Figure S1.** Distribution records of the *Parnassius glacialis*.

(a) *Parnassius glacialis*; (b) Final distribution points used for modeling.

**Figure S2.** The Pearson’s correlation analyses of 19 bioclimatic variables.

(a) Correlation matrix of the original 19 bioclimatic variables; (b) Correlation matrix of the standardized 19 bioclimatic variables.

**Figure S3.** Pearson correlation analysis among the environmental variables.

(a) Correlation matrix of the 22 candidate environmental variables (19 bioclimatic variables, elevation, HPD, and NDVI); (b) Pearson correlation between Human Population Density (HPD) and Human Footprint Index (HFP).

**Figure S4.** The final Calibration Area (CA, semi-transparent polygon) for P. glacialis in China, derived from the Minimum Convex Polygon (MCP, green dashed line) of 45 validated occurrence records (purple dots) expanded by a 50-km dispersal buffer. The inset map shows the full national boundaries including the South China Sea islands. The main map is zoomed-in on central-eastern China with a semi-transparent topographical background (Hillshade) to show how the buffer follows mountain ridgelines while avoiding deep valleys.

**Figure S5.** The receiver operating characteristic (ROC) curves of the 12 ecological niche models under the climate-only model.

**Figure S6.** The receiver operating characteristic (ROC) curves of the 12 ecological niche models under the full-variable combination (climate, elevation, HPD, and NDVI).

**Figure S7.** Receiver operating characteristic (ROC) curves of the three individual models selected for ensemble modeling under different variable combinations.

(a) GAM, (b) RF, and (c) MaxEnt based on the climate-only model; (d) MARS, (e) RF, and (f) MaxEnt based on the full-variable model (climate, elevation, HPD, and NDVI).

**Figure S8.** Boyce index under different variable combinations.

(a) Based on the climate‑only model; (b) Based on the full‑variable model (climate, elevation, HPD, and NDVI).

**Figure S9.** Response curves for the seven climatic variables included in the climate-only model, illustrating how the predicted probability of presence changes when the model is created using only that variable. The blue lines represent the average response across 10 replicate ensemble model runs; gray bands denote mean ± one standard deviation.

(a) Mean Diurnal Range (Mean of monthly (max temp - min temp)) (BIO2); (b) Temperature seasonality (standard deviation ×100) (BIO4); (c) Mean temperature of wettest quarter (BIO8); (d) Precipitation of wettest month (BIO13); (e) Precipitation of Driest Month (BIO14); (f) Precipitation Seasonality (Coefficient of Variation) (BIO15); (g) Precipitation of warmest quarter (BIO18).

**Figure S10.** Spatial uncertainty (standard deviation, SD) of the predicted suitable habitats for P. glacialis across three GCMs (BCC-CSM2-MR, IPSL-CM6A-LR, and MRI-ESM2-0) under two SSP scenarios (SSP1-2.6 and SSP5-8.5) and three time periods. (a–c) SD under SSP1-2.6 for the 2030s, 2050s, and 2070s, respectively; (d–f) SD under SSP5-8.5 for the 2030s, 2050s, and 2070s, respectively. Higher SD values (orange/red) indicate lower consensus among climate models, while lower SD values (yellow) indicate higher consensus. The inset map shows the South China Sea Islands.

**Table S1.** The occurrence records of *Parnassius glacialis* and their sources.

| **Species** | **Longitude** | **Latitude** | **Source** |
| --- | --- | --- | --- |
| *Parnassius glacialis* | 118.61113 | 32.111263 | GBIF |
| *Parnassius glacialis* | 118.77778 | 32.06167 | GBIF |
| *Parnassius glacialis* | 120.37194 | 36.09861 | GBIF |
| *Parnassius glacialis* | 114.203869 | 31.397749 | GBIF |
| *Parnassius glacialis* | 114.273278 | 31.203545 | GBIF |
| *Parnassius glacialis* | 118.562515 | 32.058987 | GBIF |
| *Parnassius glacialis* | 107.789089 | 34.047719 | GBIF |
| *Parnassius glacialis* | 119.081734 | 32.109303 | GBIF |
| *Parnassius glacialis* | 118.833382 | 32.082012 | GBIF |
| *Parnassius glacialis* | 119.122328 | 32.118143 | GBIF |
| *Parnassius glacialis* | 119.315865 | 31.795311 | GBIF |
| *Parnassius glacialis* | 117.11289 | 36.241031 | GBIF |
| *Parnassius glacialis* | 108.445445 | 33.439968 | GBIF |
| *Parnassius glacialis* | 108.45 | 33.9 | GBIF |
| *Parnassius glacialis* | 119.990294 | 31.813248 | GBIF |
| *Parnassius glacialis* | 118.822224 | 32.06851 | GBIF |
| *Parnassius glacialis* | 110.359097 | 31.491572 | GBIF |
| *Parnassius glacialis* | 118.533622 | 32.0762 | GBIF |
| *Parnassius glacialis* | 119.0812 | 32.130051 | GBIF |
| *Parnassius glacialis* | 119.454751 | 30.351713 | GBIF |
| *Parnassius glacialis* | 107.776701 | 33.838738 | GBIF |
| *Parnassius glacialis* | 104.770584 | 32.576596 | GBIF |
| *Parnassius glacialis* | 110.929586 | 31.061098 | GBIF |
| *Parnassius glacialis* | 119.440303 | 34.708167 | GBIF |
| *Parnassius glacialis* | 119.318542 | 31.788155 | GBIF |
| *Parnassius glacialis* | 98.55085 | 39.20938 | GBIF |
| *Parnassius glacialis* | 104.690707 | 32.947663 | GBIF |
| *Parnassius glacialis* | 104.376336 | 33.792735 | GBIF |
| *Parnassius glacialis* | 103.845123 | 36.065368 | GBIF |
| *Parnassius glacialis* | 104.89685 | 34.72767 | GBIF |
| *Parnassius glacialis* | 119.4 | 34.71 | GBIF |
| *Parnassius glacialis* | 115.77 | 31.17 | GBIF |
| *Parnassius glacialis* | 107.4 | 33.73 | GBIF |
| *Parnassius glacialis* | 111.66 | 33.76 | GBIF |
| *Parnassius glacialis* | 105.68 | 34.85 | GBIF |
| *Parnassius glacialis* | 118.29 | 32.28 | GBIF |
| *Parnassius glacialis* | 110.75 | 32.62 | GBIF |
| *Parnassius glacialis* | 121.73 | 37.28 | GBIF |
| *Parnassius glacialis* | 110.35 | 31.52 | GBIF |
| *Parnassius glacialis* | 113.05 | 34.49 | GBIF |
| *Parnassius glacialis* | 102.890566 | 34.989612 | GBIF |
| *Parnassius glacialis* | 103.176227 | 34.140637 | GBIF |
| *Parnassius glacialis* | 103.216053 | 34.063075 | GBIF |
| *Parnassius glacialis* | 110.388 | 31.442 | GBIF |
| *Parnassius glacialis* | 109.038 | 31.816 | GBIF |
| *Parnassius glacialis* | 110.39 | 31.44 | Literature |
| *Parnassius glacialis* | 110.84 | 32.07 | Literature |

Note: Records providing only province-level locality names (e.g., Yunnan and Gansu) were strictly excluded due to unacceptable spatial uncertainty (>20 km). Consequently, only 2 of the 6 literature-derived records, which had precise coordinates directly provided in the original publications, were retained, resulting in a total of 47 raw records (45 from GBIF and 2 from literature) before spatial thinning.

**Table S2.** The occurrence records used in the ensemble models.

| **species** | **Longitude** | **Latitude** |
| --- | --- | --- |
| *Parnassius glacialis* | 118.61113 | 32.111263 |
| *Parnassius glacialis* | 118.77778 | 32.06167 |
| *Parnassius glacialis* | 120.37194 | 36.09861 |
| *Parnassius glacialis* | 114.203869 | 31.397749 |
| *Parnassius glacialis* | 119.4 | 34.71 |
| *Parnassius glacialis* | 115.77 | 31.17 |
| *Parnassius glacialis* | 107.4 | 33.73 |
| *Parnassius glacialis* | 111.66 | 33.76 |
| *Parnassius glacialis* | 105.68 | 34.85 |
| *Parnassius glacialis* | 118.29 | 32.28 |
| *Parnassius glacialis* | 110.75 | 32.62 |
| *Parnassius glacialis* | 121.73 | 37.28 |
| *Parnassius glacialis* | 110.35 | 31.52 |
| *Parnassius glacialis* | 113.05 | 34.49 |
| *Parnassius glacialis* | 102.890566 | 34.989612 |
| *Parnassius glacialis* | 103.176227 | 34.140637 |
| *Parnassius glacialis* | 103.216053 | 34.063075 |
| *Parnassius glacialis* | 110.388 | 31.442 |
| *Parnassius glacialis* | 109.038 | 31.816 |
| *Parnassius glacialis* | 107.789089 | 34.047719 |
| *Parnassius glacialis* | 119.081734 | 32.109303 |
| *Parnassius glacialis* | 118.833382 | 32.082012 |
| *Parnassius glacialis* | 119.122328 | 32.118143 |
| *Parnassius glacialis* | 119.440303 | 34.708167 |
| *Parnassius glacialis* | 119.318542 | 31.788155 |
| *Parnassius glacialis* | 98.55085 | 39.20938 |
| *Parnassius glacialis* | 104.690707 | 32.947663 |
| *Parnassius glacialis* | 104.376336 | 33.792735 |
| *Parnassius glacialis* | 103.845123 | 36.065368 |
| *Parnassius glacialis* | 104.89685 | 34.72767 |
| *Parnassius glacialis* | 114.273278 | 31.203545 |
| *Parnassius glacialis* | 118.562515 | 32.058987 |
| *Parnassius glacialis* | 119.315865 | 31.795311 |
| *Parnassius glacialis* | 117.11289 | 36.241031 |
| *Parnassius glacialis* | 108.445445 | 33.439968 |
| *Parnassius glacialis* | 108.45 | 33.9 |
| *Parnassius glacialis* | 119.990294 | 31.813248 |
| *Parnassius glacialis* | 118.822224 | 32.06851 |
| *Parnassius glacialis* | 110.359097 | 31.491572 |
| *Parnassius glacialis* | 118.533622 | 32.0762 |
| *Parnassius glacialis* | 119.0812 | 32.130051 |
| *Parnassius glacialis* | 119.454751 | 30.351713 |
| *Parnassius glacialis* | 107.776701 | 33.838738 |
| *Parnassius glacialis* | 104.770584 | 32.576596 |
| *Parnassius glacialis* | 110.929586 | 31.061098 |

**Table S3.** The environmental variables considered in this study.

| **Variable type** | **Variable** | **Description** |
| --- | --- | --- |
| Bioclimatic Variables | BIO1 | Annual mean temperature  ($BIO1={\sum Monthly Mean Temperatures}/{12}$) (℃) |
|  | **BIO2** | Mean diurnal range  $(BIO2={\sum\left( Monthly Max Temp-Monthly Min Temp \right)}/{12})$ (℃) |
|  | BIO3 | Isothermality  $(BIO3={(BIO2}/{BIO7})\times100)$ |
|  | **BIO4** | Temperature seasonality  $(BIO4=\left( standard deviation\times100 \right))$ |
|  | BIO5 | Max temperature of warmest month  $\left( BIO5=Maximum Monthly Max Temperature \right)$(℃) |
|  | BIO6 | Min temperature of coldest month  $\left( BIO6=Minimum Monthly Min Temperature \right)$(℃) |
|  | BIO7 | Annual temperature range  $(BIO7=BIO5-BIO6)$ (℃) |
|  | **BIO8** | Mean temperature of the wettest quarter  $(BIO8=Mean Temperature of the Wettest 3-Month Period)$ (℃) |
|  | BIO9 | Mean temperature of driest quarter  $(BIO9=Mean Temperature of the Driest 3-Month Period)$ (℃) |
|  | BIO10 | Mean temperature of warmest quarter  $(BIO10=Mean Temperature of the Warmest 3-Month Period)$ (℃) |
|  | BIO11 | Mean temperature of coldest quarter  $(BIO11=Mean Temperature of the Coldest 3-Month Period)$ (℃) |
|  | BIO12 | Annual precipitation  $(BIO12=\sum Monthly Precipitation)$(mm) |
|  | **BIO13** | Precipitation of the wettest month  $(BIO13=Maximum Monthly Precipitation)$(mm) |
|  | **BIO14** | Precipitation of the driest month  $(BIO14=Minimum Monthly Precipitation)$ (mm) |
|  | **BIO15** | Precipitation seasonality  $(BIO15=\left( {Standard Deviation of Monthly Precipitation}/{Mean Monthly Precipitation} \right)\times100 \left( coefficient of variation \right))$ (mm) |
|  | BIO16 | Precipitation of wettest quarter  $(BIO16=Total Precipitation of the Wettest 3-Month Period)$ (mm) |
|  | BIO17 | Precipitation of driest quarter  $(BIO17=Total Precipitation of the Driest 3-Month Period)$ (mm) |
|  | **BIO18** | Precipitation of the warmest quarter  $(BIO18=Total Precipitation of the Warmest 3-Month Period)$ (mm) |
|  | BIO19 | Precipitation of coldest quarter  $(BIO19=Total Precipitation of the Coldest 3-Month Period)$(mm) |
| Elevation | **elev** | Ground height above sea level (m) |
| Human Population Density | **HPD** | A measurement of the number of people in an area  $(\mathrm{HPD}={Total Population}/{Land Area})$ (people/km²) |
| Normalized Difference Vegetation Index | **NDVI** | NDVI is one of the important parameters reflecting the growth and nutritional information of crops  $(NDVI=(NIR-Red)/(NIR+Red))$ |

Note: The 19 bioclimatic variables and elevation data were extracted from WorldClim v2.1 (http://www.worldclim.org/) at a spatial resolution of 2.5 arc-minutes. Human Population Density (HPD) data were obtained from the Gridded Population of the World Version 4 (GPWv4) for the year 2000, and the Normalized Difference Vegetation Index (NDVI) data were obtained from the MODIS MOD13Q1 Version 6 product for the year 2023. All layers were resampled to a 2.5 arc-minutes resolution using nearest-neighbor resampling. Only climatic variables were used for future climate scenario projections.

**Table S4.** Summary of absence/background types and sampling parameters for each algorithm.

| **Algorithm** | **Absence/Background Type** | **Number of Points** | | **Sampling Method** | **Exclusion Buffer** |
| --- | --- | --- | --- | --- | --- |
| MaxEnt | Background | 10,000 | Random | | No |
| MaxLike | Background | 10,000 | Random | | No |
| RF | Pseudo-absence | 1,000 | Random | | No |
| GAM | Pseudo-absence | 1,000 | Random | | No |
| GLM | Pseudo-absence | 1,000 | Random | | No |
| CART | Pseudo-absence | 1,000 | Random | | No |
| FDA | Pseudo-absence | 1,000 | Random | | No |
| MARS | Pseudo-absence | 1,000 | Random | | No |
| SVM | Pseudo-absence | 1,000 | Random | | No |
| Domain | Pseudo-absence | 1,000 | Random | | No |
| GLMNET | Pseudo-absence | 1,000 | Random | | No |
| MDA | Pseudo-absence | 1,000 | Random | | No |

Note: Both background and pseudo-absence points were randomly sampled within the delineated calibration area (CA). MaxEnt and MaxLike operate under a presence-background framework, using 10,000 background points. The remaining 10 algorithms operate under a presence-pseudoabsence framework, using 1,000 pseudo-absence points. No exclusion buffers were applied around occurrence records. The generated point sets were kept fixed across all 10 repeated random sub-sampling (75:25 holdout) runs to ensure fair comparison among algorithms, with only the training (75%) and testing (25%) data partitions varying across iterations.

**Table S5.** Summary of default hyperparameter configurations for each algorithm used in the 'sdm' package.

| **Algorithm** | **Hyperparameter** | **Setting** | **Source** |
| --- | --- | --- | --- |
| MaxEnt | regularization multiplier | 1 (default) | sdm package default |
| MaxEnt | feature classes | auto (L, Q, P, T, H) | sdm package default |
| MaxEnt | convergence threshold | 1×10⁻⁵ | sdm package default |
| MaxEnt | maximum iterations | 1,000 | sdm package default |
| RF | ntree (number of trees) | 500 | sdm package default |
| RF | mtry | sqrt(nvars) | sdm package default |
| GAM | smoothness | penalized splines (default) | sdm package default |
| GAM | basis dimension (k) | 10 | sdm package default |
| SVM | kernel | radial | sdm package default |
| SVM | cost | 1 | sdm package default |
| SVM | gamma | 1/nvars | sdm package default |
| MARS | interaction degree | 1 | sdm package default |
| MARS | penalty | 2 | sdm package default |
| MARS | maximum basis functions | 21 | sdm package default |
| GLMNET | alpha (elastic-net mixing) | 1 (LASSO) | sdm package default |
| GLMNET | lambda | auto-selected via CV | sdm package default |
| GLM | (all) | R default | stats package default |
| CART | (all) | R default | rpart package default |
| FDA | (all) | R default | mda package default |
| Domain | (all) | R default | sdm package default |
| MDA | (all) | R default | mda package default |
| MaxLike | method | BFGS | maxlike package default |
| MaxLike | link | logit | maxlike package default |
| MaxLike | start | Automatic | maxlike package default |
| MaxLike | iterlim | 500 | maxlike package default |

Note: All 12 algorithms were implemented using the default hyperparameter configurations provided by the 'sdm' package (Naimi and Araujo 2016), without additional systematic tuning. This decision was based on three considerations: (i) with only 45 occurrence records (a small-sample scenario), further partitioning the data into separate tuning, training, and testing sets would substantially reduce training set representativeness and exacerbate overfitting risks; (ii) the default parameters have been validated across a wide range of ecological modeling applications and generally provide stable and reasonable performance; and (iii) the final ensemble model employs a weighted averaging strategy that can effectively buffer the uncertainty introduced by hyperparameter choices in individual algorithms.

**Table S6.** Sørensen and Jaccard Similarity Indices between Current and Future Habitat Suitability Maps.

| Climate Scenario | Period | Sørensen Index | Jaccard Index |
| --- | --- | --- | --- |
| SSP1-2.6 | 2030s | 0.77 | 0.59 |
| SSP5-8.5 | 2030s | 0.77 | 0.58 |
| SSP1-2.6 | 2050s | 0.79 | 0.60 |
| SSP5-8.5 | 2050s | 0.77 | 0.58 |
| SSP1-2.6 | 2070s | 0.78 | 0.59 |
| SSP5-8.5 | 2070s | 0.78 | 0.59 |

Note: These indices are not validation statistics for future scenarios. They were calculated as map similarity indices to quantify the spatial overlap of suitable habitats between current and future projections using the data-driven threshold (MTSPS = 0.2).

**Table S7.** The suitable areas of *Parnassius glacialis* under current (×10⁴ km²).

| **Areas** | **Low**  **suitability**  **(0.2**-**0.4)** | **Medium suitability**  **(0.4**-**0.6)** | **High suitability**  **(0.6**-**1)** | **Suitable habitat**  **(0.2**-**1)** |
| --- | --- | --- | --- | --- |
| Climate | 63.50  (6.61%) | 38.12  (3.97%) | 6.74  (0.70%) | 108.35 (11.29%) |
| Climate, elevation,  HPD and NDVI | 64.09  (6.68%) | 28.17  (2.93%) | 8.48  (0.88%) | 100.74 (10.49%) |

Note: The percentages in parentheses indicate the proportion of suitable areas occupied by different levels of habitat suitability relative to the total land area of China. The model incorporating climate, elevation, HPD, and NDVI was constructed solely to characterize current potential distribution with additional ecological constraints.

**Table S8.** The suitable areas of *Parnassius glacialis* under climate conditions (×10⁴ km²).

| **Areas** | **Low**  **suitability**  **(0.2**-**0.4)** | **Medium suitability**  **(0.4**-**0.6)** | **High suitability**  **(0.6**-**1)** | **Suitable habitat**  **(0.2**-**1)** |
| --- | --- | --- | --- | --- |
| current | 63.50  (6.61%) | 38.12  (3.97%) | 6.74  (0.70%) | 108.35 (11.29%) |
| 2030s SSP126 | 101.10  (10.53%) | 5.78  (0.60%) | 0.00  (0.00%) | 106.88 (11.13%) |
| 2030s SSP585 | 101.35  (10.56%) | 5.51  (0.57%) | 0.00  (0.00%) | 106.86 (11.13%) |
| 2050s SSP126 | 101.81  (10.61%) | 5.80  (0.60%) | 0.00  (0.00%) | 107.60 (11.21%) |
| 2050s SSP585 | 103.35  (10.77%) | 3.88  (0.40%) | 0.00  (0.00%) | 107.23 (11.17%) |
| 2070s SSP126 | 98.56  (10.27%) | 4.86  (0.51%) | 0.00  (0.00%) | 103.41 (10.77%) |
| 2070s SSP585 | 101.84  (10.61%) | 1.83  (0.19%) | 0.00  (0.00%) | 103.67 (10.80%) |

Note: The percentages in parentheses indicate the proportion of suitable areas occupied by different levels of habitat suitability relative to the total land area of China.

**Table S9.** Changes of spatial distribution pattern of *Parnassius glacialis* under different climate conditions.

| **Periods** | **Habitat area/×10^4^Km^2^** | **Expansion/**  **×10^4^Km^2^** | **Contraction/**  **×10^4^Km^2^** | **Stable/**  **×10^4^Km^2^** | **Pecentage expansion** | **Pecentage Contraction** | **Overall range change** |
| --- | --- | --- | --- | --- | --- | --- | --- |
| 1970-2000 | 108.35 |  |  |  |  |  |  |
| 2030s SSP126 | 106.88 | 51.14 | 49.41 | 63.3 | 47.20% | 45.60% | -1.36% |
| 2050s SSP126 | 107.6 | 50.22 | 47.78 | 64.94 | 46.35% | 44.10% | -0.69% |
| 2070s SSP126 | 103.41 | 48.15 | 50.19 | 62.52 | 44.44% | 46.32% | -4.56% |
| 2030s SSP585 | 106.86 | 52.24 | 50.51 | 62.21 | 48.21% | 46.62% | -1.38% |
| 2050s SSP585 | 107.23 | 50.58 | 48.57 | 110.89 | 46.68% | 44.83% | -1.03% |
| 2070s SSP585 | 103.67 | 48.43 | 50.24 | 106.97 | 44.70% | 46.37% | -4.32% |

**Table S10.** Centroid shifting distance and speed of *Parnassius glacialis* in different periods

| Time Interval | Centroid Shifting Distance (km) | Centroid Shifting Speed (km/year) |
| --- | --- | --- |
| Current – 2030s SSP1-2.6 | 356 | 11.87 |
| Current – 2030s SSP5-8.5 | 357 | 11.90 |
| 2030s SSP1-2.6 – 2050s SSP1-2.6 | 17 | 0.85 |
| 2030s SSP5-8.5 – 2050s SSP5-8.5 | 17 | 0.85 |
| 2050s SSP1-2.6 – 2070s SSP1-2.6 | 26 | 1.30 |
| 2050s SSP5-8.5 – 2070s SSP5-8.5 | 44 | 2.20 |

Note: The centroid coordinates of suitable habitats for each period were extracted using the "Centroid Changes (Lines)" tool in ArcGIS 10.4. To eliminate calculation errors in migration distance caused by geographic coordinate systems, all centroid datasets were reprojected to the Albers Equal Area Conic coordinate system (WGS 1984 datum) prior to calculating distances. The shifting speed was determined by dividing the centroid shifting distance by the time interval. Specifically, the interval between "Current" (representing the baseline climate of 1970–2000) and the "2030s" (representing 2021–2040) was set to 30 years (centered at 1985 vs. 2030), while the intervals between subsequent periods (e.g., 2030s to 2050s, 2050s to 2070s) were set to 20 years.

**Table S11.** Elevation changes of the geographic centroid of *Parnassius glacialis* suitable habitats across different periods.

| **Scenario** | **Period** | **Centroid Longitude (°E)** | **Centroid Latitude (°N)** | **Centroid Elevation (m)** | **Elevation Change (m)** |
| --- | --- | --- | --- | --- | --- |
| current | current | 110.57 | 32.75 | 354 | — |
| SSP1-2.6 | 2030s | 112.64 | 30.04 | 27 | −327 |
| SSP1-2.6 | 2050s | 112.46 | 30.08 | 30 | −324 |
| SSP1-2.6 | 2070s | 112.73 | 30.14 | 27 | −327 |
| SSP5-8.5 | 2030s | 112.45 | 29.93 | 30 | −324 |
| SSP5-8.5 | 2050s | 112.37 | 30.07 | 31 | −323 |
| SSP5-8.5 | 2070s | 111.9 | 30.11 | 36 | −318 |

Note: The elevation values were extracted directly from the WorldClim v2.1 elevation layer at the centroid coordinates of current and future suitable habitats using the 'Extract Multi Values to Points' tool in ArcGIS 10.4.

**Table S12.** BIO2 values at the 45 occurrence points under current climate conditions.

| **species** | **Longitude** | **Latitude** | **RASTERVALU** |
| --- | --- | --- | --- |
| *Parnassius glacialis* | 118.61113 | 32.111263 | 8.238666534 |
| *Parnassius glacialis* | 118.77778 | 32.06167 | 8.247333527 |
| *Parnassius glacialis* | 120.37194 | 36.09861 | 7.161000252 |
| *Parnassius glacialis* | 114.203869 | 31.397749 | 8.315333366 |
| *Parnassius glacialis* | 114.273278 | 31.203545 | 7.982333183 |
| *Parnassius glacialis* | 118.562515 | 32.058987 | 8.207333565 |
| *Parnassius glacialis* | 107.789089 | 34.047719 | 7.607666492 |
| *Parnassius glacialis* | 119.081734 | 32.109303 | 8.125 |
| *Parnassius glacialis* | 118.833382 | 32.082012 | 8.232666969 |
| *Parnassius glacialis* | 119.122328 | 32.118143 | 8.081000328 |
| *Parnassius glacialis* | 119.315865 | 31.795311 | 7.893333435 |
| *Parnassius glacialis* | 117.11289 | 36.241031 | 9.295000076 |
| *Parnassius glacialis* | 108.445445 | 33.439968 | 7.84800005 |
| *Parnassius glacialis* | 108.45 | 33.9 | 7.639999866 |
| *Parnassius glacialis* | 119.990294 | 31.813248 | 7.827999592 |
| *Parnassius glacialis* | 118.822224 | 32.06851 | 8.302332878 |
| *Parnassius glacialis* | 110.359097 | 31.491572 | 8.369333267 |
| *Parnassius glacialis* | 118.533622 | 32.0762 | 8.170666695 |
| *Parnassius glacialis* | 119.0812 | 32.130051 | 8.122333527 |
| *Parnassius glacialis* | 119.454751 | 30.351713 | 7.268000126 |
| *Parnassius glacialis* | 107.776701 | 33.838738 | 7.969333172 |
| *Parnassius glacialis* | 104.770584 | 32.576596 | 8.392333031 |
| *Parnassius glacialis* | 110.929586 | 31.061098 | 8 |
| *Parnassius glacialis* | 119.440303 | 34.708167 | 8.036231995 |
| *Parnassius glacialis* | 119.318542 | 31.788155 | 7.950333595 |
| *Parnassius glacialis* | 98.55085 | 39.20938 | 11.14000034 |
| *Parnassius glacialis* | 104.690707 | 32.947663 | 9.363666534 |
| *Parnassius glacialis* | 104.376336 | 33.792735 | 9.895667076 |
| *Parnassius glacialis* | 103.845123 | 36.065368 | 12.14566708 |
| *Parnassius glacialis* | 104.89685 | 34.72767 | 10.24233341 |
| *Parnassius glacialis* | 119.4 | 34.71 | 8.0625 |
| *Parnassius glacialis* | 115.77 | 31.17 | 8.520000458 |
| *Parnassius glacialis* | 107.4 | 33.73 | 8.096666336 |
| *Parnassius glacialis* | 111.66 | 33.76 | 10.899333 |
| *Parnassius glacialis* | 105.68 | 34.85 | 10.56066704 |
| *Parnassius glacialis* | 118.29 | 32.28 | 8.470666885 |
| *Parnassius glacialis* | 110.75 | 32.62 | 10.09899998 |
| *Parnassius glacialis* | 121.73 | 37.28 | 6.290333271 |
| *Parnassius glacialis* | 110.35 | 31.52 | 8.476666451 |
| *Parnassius glacialis* | 113.05 | 34.49 | 10.22900009 |
| *Parnassius glacialis* | 102.890566 | 34.989612 | 14.18099976 |
| *Parnassius glacialis* | 103.176227 | 34.140637 | 11.87233353 |
| *Parnassius glacialis* | 103.216053 | 34.063075 | 12.17033386 |
| *Parnassius glacialis* | 110.388 | 31.442 | 8.371333122 |
| *Parnassius glacialis* | 109.038 | 31.816 | 8.076000214 |

**Table S13.** BIO14 values at the 45 occurrence points under current climate conditions.

| **species** | **Longitude** | **Latitude** | **RASTERVALU** |
| --- | --- | --- | --- |
| *Parnassius glacialis* | 118.61113 | 32.111263 | 25 |
| *Parnassius glacialis* | 118.77778 | 32.06167 | 29 |
| *Parnassius glacialis* | 120.37194 | 36.09861 | 13 |
| *Parnassius glacialis* | 114.203869 | 31.397749 | 21 |
| *Parnassius glacialis* | 114.273278 | 31.203545 | 22 |
| *Parnassius glacialis* | 118.562515 | 32.058987 | 25 |
| *Parnassius glacialis* | 107.789089 | 34.047719 | 5 |
| *Parnassius glacialis* | 119.081734 | 32.109303 | 24 |
| *Parnassius glacialis* | 118.833382 | 32.082012 | 26 |
| *Parnassius glacialis* | 119.122328 | 32.118143 | 24 |
| *Parnassius glacialis* | 119.315865 | 31.795311 | 27 |
| *Parnassius glacialis* | 117.11289 | 36.241031 | 6 |
| *Parnassius glacialis* | 108.445445 | 33.439968 | 8 |
| *Parnassius glacialis* | 108.45 | 33.9 | 6 |
| *Parnassius glacialis* | 119.990294 | 31.813248 | 37 |
| *Parnassius glacialis* | 118.822224 | 32.06851 | 28 |
| *Parnassius glacialis* | 110.359097 | 31.491572 | 20 |
| *Parnassius glacialis* | 118.533622 | 32.0762 | 26 |
| *Parnassius glacialis* | 119.0812 | 32.130051 | 26 |
| *Parnassius glacialis* | 119.454751 | 30.351713 | 40 |
| *Parnassius glacialis* | 107.776701 | 33.838738 | 6 |
| *Parnassius glacialis* | 104.770584 | 32.576596 | 4 |
| *Parnassius glacialis* | 110.929586 | 31.061098 | 24 |
| *Parnassius glacialis* | 119.440303 | 34.708167 | 14 |
| *Parnassius glacialis* | 119.318542 | 31.788155 | 27 |
| *Parnassius glacialis* | 98.55085 | 39.20938 | 4 |
| *Parnassius glacialis* | 104.690707 | 32.947663 | 2 |
| *Parnassius glacialis* | 104.376336 | 33.792735 | 3 |
| *Parnassius glacialis* | 103.845123 | 36.065368 | 2 |
| *Parnassius glacialis* | 104.89685 | 34.72767 | 2 |
| *Parnassius glacialis* | 119.4 | 34.71 | 14 |
| *Parnassius glacialis* | 115.77 | 31.17 | 29 |
| *Parnassius glacialis* | 107.4 | 33.73 | 6 |
| *Parnassius glacialis* | 111.66 | 33.76 | 8 |
| *Parnassius glacialis* | 105.68 | 34.85 | 3 |
| *Parnassius glacialis* | 118.29 | 32.28 | 27 |
| *Parnassius glacialis* | 110.75 | 32.62 | 13 |
| *Parnassius glacialis* | 121.73 | 37.28 | 11 |
| *Parnassius glacialis* | 110.35 | 31.52 | 20 |
| *Parnassius glacialis* | 113.05 | 34.49 | 7 |
| *Parnassius glacialis* | 102.890566 | 34.989612 | 1 |
| *Parnassius glacialis* | 103.176227 | 34.140637 | 1 |
| *Parnassius glacialis* | 103.216053 | 34.063075 | 1 |
| *Parnassius glacialis* | 110.388 | 31.442 | 19 |
| *Parnassius glacialis* | 109.038 | 31.816 | 15 |

**Table S14**. BIO8 values at the 45 occurrence points under current climate conditions.

| **species** | **Longitude** | **Latitude** | **RASTERVALU** |
| --- | --- | --- | --- |
| *Parnassius glacialis* | 118.61113 | 32.111263 | 26.11333275 |
| *Parnassius glacialis* | 118.77778 | 32.06167 | 27.07600021 |
| *Parnassius glacialis* | 120.37194 | 36.09861 | 24.07266617 |
| *Parnassius glacialis* | 114.203869 | 31.397749 | 24.21800041 |
| *Parnassius glacialis* | 114.273278 | 31.203545 | 24.00866699 |
| *Parnassius glacialis* | 118.562515 | 32.058987 | 25.98266602 |
| *Parnassius glacialis* | 107.789089 | 34.047719 | 16.22200012 |
| *Parnassius glacialis* | 119.081734 | 32.109303 | 26.22333336 |
| *Parnassius glacialis* | 118.833382 | 32.082012 | 26.39066696 |
| *Parnassius glacialis* | 119.122328 | 32.118143 | 25.93199921 |
| *Parnassius glacialis* | 119.315865 | 31.795311 | 26.1780014 |
| *Parnassius glacialis* | 117.11289 | 36.241031 | 22.75266647 |
| *Parnassius glacialis* | 108.445445 | 33.439968 | 16.4746666 |
| *Parnassius glacialis* | 108.45 | 33.9 | 14.72200012 |
| *Parnassius glacialis* | 119.990294 | 31.813248 | 27.09399986 |
| *Parnassius glacialis* | 118.822224 | 32.06851 | 26.93666649 |
| *Parnassius glacialis* | 110.359097 | 31.491572 | 17.29133415 |
| *Parnassius glacialis* | 118.533622 | 32.0762 | 25.87333298 |
| *Parnassius glacialis* | 119.0812 | 32.130051 | 26.22866631 |
| *Parnassius glacialis* | 119.454751 | 30.351713 | 19.48666763 |
| *Parnassius glacialis* | 107.776701 | 33.838738 | 15.7213335 |
| *Parnassius glacialis* | 104.770584 | 32.576596 | 17.55066681 |
| *Parnassius glacialis* | 110.929586 | 31.061098 | 20.02733231 |
| *Parnassius glacialis* | 119.440303 | 34.708167 | 24.6427536 |
| *Parnassius glacialis* | 119.318542 | 31.788155 | 26.17333221 |
| *Parnassius glacialis* | 98.55085 | 39.20938 | 1.799999952 |
| *Parnassius glacialis* | 104.690707 | 32.947663 | 20.49533272 |
| *Parnassius glacialis* | 104.376336 | 33.792735 | 13.69999981 |
| *Parnassius glacialis* | 103.845123 | 36.065368 | 19.82999992 |
| *Parnassius glacialis* | 104.89685 | 34.72767 | 16.49600029 |
| *Parnassius glacialis* | 119.4 | 34.71 | 23.98749924 |
| *Parnassius glacialis* | 115.77 | 31.17 | 23.68133354 |
| *Parnassius glacialis* | 107.4 | 33.73 | 18.51933289 |
| *Parnassius glacialis* | 111.66 | 33.76 | 22.19466591 |
| *Parnassius glacialis* | 105.68 | 34.85 | 19.13466644 |
| *Parnassius glacialis* | 118.29 | 32.28 | 25.95466614 |
| *Parnassius glacialis* | 110.75 | 32.62 | 25.29400063 |
| *Parnassius glacialis* | 121.73 | 37.28 | 21.62333298 |
| *Parnassius glacialis* | 110.35 | 31.52 | 16.95999908 |
| *Parnassius glacialis* | 113.05 | 34.49 | 22.19400024 |
| *Parnassius glacialis* | 102.890566 | 34.989612 | 11.58399963 |
| *Parnassius glacialis* | 103.176227 | 34.140637 | 11.85200024 |
| *Parnassius glacialis* | 103.216053 | 34.063075 | 13.49400043 |
| *Parnassius glacialis* | 110.388 | 31.442 | 19.40333366 |
| *Parnassius glacialis* | 109.038 | 31.816 | 19.54599953 |

**Table S15.** BIO13 values at the 45 occurrence points under current climate conditions.

| **species** | **Longitude** | **Latitude** | **RASTERVALU** |
| --- | --- | --- | --- |
| *Parnassius glacialis* | 118.61113 | 32.111263 | 203 |
| *Parnassius glacialis* | 118.77778 | 32.06167 | 211 |
| *Parnassius glacialis* | 120.37194 | 36.09861 | 161 |
| *Parnassius glacialis* | 114.203869 | 31.397749 | 200 |
| *Parnassius glacialis* | 114.273278 | 31.203545 | 198 |
| *Parnassius glacialis* | 118.562515 | 32.058987 | 201 |
| *Parnassius glacialis* | 107.789089 | 34.047719 | 131 |
| *Parnassius glacialis* | 119.081734 | 32.109303 | 194 |
| *Parnassius glacialis* | 118.833382 | 32.082012 | 201 |
| *Parnassius glacialis* | 119.122328 | 32.118143 | 193 |
| *Parnassius glacialis* | 119.315865 | 31.795311 | 172 |
| *Parnassius glacialis* | 117.11289 | 36.241031 | 234 |
| *Parnassius glacialis* | 108.445445 | 33.439968 | 150 |
| *Parnassius glacialis* | 108.45 | 33.9 | 140 |
| *Parnassius glacialis* | 119.990294 | 31.813248 | 179 |
| *Parnassius glacialis* | 118.822224 | 32.06851 | 205 |
| *Parnassius glacialis* | 110.359097 | 31.491572 | 211 |
| *Parnassius glacialis* | 118.533622 | 32.0762 | 201 |
| *Parnassius glacialis* | 119.0812 | 32.130051 | 199 |
| *Parnassius glacialis* | 119.454751 | 30.351713 | 241 |
| *Parnassius glacialis* | 107.776701 | 33.838738 | 136 |
| *Parnassius glacialis* | 104.770584 | 32.576596 | 152 |
| *Parnassius glacialis* | 110.929586 | 31.061098 | 214 |
| *Parnassius glacialis* | 119.440303 | 34.708167 | 222 |
| *Parnassius glacialis* | 119.318542 | 31.788155 | 170 |
| *Parnassius glacialis* | 98.55085 | 39.20938 | 102 |
| *Parnassius glacialis* | 104.690707 | 32.947663 | 139 |
| *Parnassius glacialis* | 104.376336 | 33.792735 | 114 |
| *Parnassius glacialis* | 103.845123 | 36.065368 | 76 |
| *Parnassius glacialis* | 104.89685 | 34.72767 | 106 |
| *Parnassius glacialis* | 119.4 | 34.71 | 232 |
| *Parnassius glacialis* | 115.77 | 31.17 | 231 |
| *Parnassius glacialis* | 107.4 | 33.73 | 136 |
| *Parnassius glacialis* | 111.66 | 33.76 | 148 |
| *Parnassius glacialis* | 105.68 | 34.85 | 108 |
| *Parnassius glacialis* | 118.29 | 32.28 | 203 |
| *Parnassius glacialis* | 110.75 | 32.62 | 172 |
| *Parnassius glacialis* | 121.73 | 37.28 | 186 |
| *Parnassius glacialis* | 110.35 | 31.52 | 209 |
| *Parnassius glacialis* | 113.05 | 34.49 | 162 |
| *Parnassius glacialis* | 102.890566 | 34.989612 | 111 |
| *Parnassius glacialis* | 103.176227 | 34.140637 | 115 |
| *Parnassius glacialis* | 103.216053 | 34.063075 | 111 |
| *Parnassius glacialis* | 110.388 | 31.442 | 209 |
| *Parnassius glacialis* | 109.038 | 31.816 | 191 |

**Table S16.** BIO15 values at the 45 occurrence points under current climate conditions.

| **species** | **Longitude** | **Latitude** | **RASTERVALU** |
| --- | --- | --- | --- |
| *Parnassius glacialis* | 118.61113 | 32.111263 | 60.52206039 |
| *Parnassius glacialis* | 118.77778 | 32.06167 | 59.49874878 |
| *Parnassius glacialis* | 120.37194 | 36.09861 | 86.70890045 |
| *Parnassius glacialis* | 114.203869 | 31.397749 | 58.1008606 |
| *Parnassius glacialis* | 114.273278 | 31.203545 | 56.95184326 |
| *Parnassius glacialis* | 118.562515 | 32.058987 | 59.65143585 |
| *Parnassius glacialis* | 107.789089 | 34.047719 | 75.49215698 |
| *Parnassius glacialis* | 119.081734 | 32.109303 | 59.48751068 |
| *Parnassius glacialis* | 118.833382 | 32.082012 | 59.15578461 |
| *Parnassius glacialis* | 119.122328 | 32.118143 | 59.04100418 |
| *Parnassius glacialis* | 119.315865 | 31.795311 | 53.75915909 |
| *Parnassius glacialis* | 117.11289 | 36.241031 | 111.628273 |
| *Parnassius glacialis* | 108.445445 | 33.439968 | 70.66721344 |
| *Parnassius glacialis* | 108.45 | 33.9 | 73.01570129 |
| *Parnassius glacialis* | 119.990294 | 31.813248 | 55.01874924 |
| *Parnassius glacialis* | 118.822224 | 32.06851 | 58.86659622 |
| *Parnassius glacialis* | 110.359097 | 31.491572 | 62.90385056 |
| *Parnassius glacialis* | 118.533622 | 32.0762 | 59.438591 |
| *Parnassius glacialis* | 119.0812 | 32.130051 | 60.18774796 |
| *Parnassius glacialis* | 119.454751 | 30.351713 | 49.382061 |
| *Parnassius glacialis* | 107.776701 | 33.838738 | 73.96623993 |
| *Parnassius glacialis* | 104.770584 | 32.576596 | 83.86179352 |
| *Parnassius glacialis* | 110.929586 | 31.061098 | 60.96788025 |
| *Parnassius glacialis* | 119.440303 | 34.708167 | 93.59492493 |
| *Parnassius glacialis* | 119.318542 | 31.788155 | 53.24664307 |
| *Parnassius glacialis* | 98.55085 | 39.20938 | 101.5250168 |
| *Parnassius glacialis* | 104.690707 | 32.947663 | 88.001091 |
| *Parnassius glacialis* | 104.376336 | 33.792735 | 80.34159851 |
| *Parnassius glacialis* | 103.845123 | 36.065368 | 88.27348328 |
| *Parnassius glacialis* | 104.89685 | 34.72767 | 83.18112946 |
| *Parnassius glacialis* | 119.4 | 34.71 | 94.6384964 |
| *Parnassius glacialis* | 115.77 | 31.17 | 57.34159088 |
| *Parnassius glacialis* | 107.4 | 33.73 | 74.32144165 |
| *Parnassius glacialis* | 111.66 | 33.76 | 71.08937836 |
| *Parnassius glacialis* | 105.68 | 34.85 | 80.81633759 |
| *Parnassius glacialis* | 118.29 | 32.28 | 62.36143494 |
| *Parnassius glacialis* | 110.75 | 32.62 | 66.63375854 |
| *Parnassius glacialis* | 121.73 | 37.28 | 95.729599 |
| *Parnassius glacialis* | 110.35 | 31.52 | 63.00846863 |
| *Parnassius glacialis* | 113.05 | 34.49 | 82.37381744 |
| *Parnassius glacialis* | 102.890566 | 34.989612 | 89.13040161 |
| *Parnassius glacialis* | 103.176227 | 34.140637 | 85.78583527 |
| *Parnassius glacialis* | 103.216053 | 34.063075 | 85.37091064 |
| *Parnassius glacialis* | 110.388 | 31.442 | 63.53768539 |
| *Parnassius glacialis* | 109.038 | 31.816 | 64.84143066 |

**Table S17.** BIO18 values at the 45 occurrence points under current climate conditions.

| **species** | **Longitude** | **Latitude** | **RASTERVALU** |
| --- | --- | --- | --- |
| *Parnassius glacialis* | 118.61113 | 32.111263 | 472 |
| *Parnassius glacialis* | 118.77778 | 32.06167 | 492 |
| *Parnassius glacialis* | 120.37194 | 36.09861 | 388 |
| *Parnassius glacialis* | 114.203869 | 31.397749 | 491 |
| *Parnassius glacialis* | 114.273278 | 31.203545 | 500 |
| *Parnassius glacialis* | 118.562515 | 32.058987 | 473 |
| *Parnassius glacialis* | 107.789089 | 34.047719 | 326 |
| *Parnassius glacialis* | 119.081734 | 32.109303 | 468 |
| *Parnassius glacialis* | 118.833382 | 32.082012 | 478 |
| *Parnassius glacialis* | 119.122328 | 32.118143 | 470 |
| *Parnassius glacialis* | 119.315865 | 31.795311 | 460 |
| *Parnassius glacialis* | 117.11289 | 36.241031 | 487 |
| *Parnassius glacialis* | 108.445445 | 33.439968 | 369 |
| *Parnassius glacialis* | 108.45 | 33.9 | 352 |
| *Parnassius glacialis* | 119.990294 | 31.813248 | 464 |
| *Parnassius glacialis* | 118.822224 | 32.06851 | 482 |
| *Parnassius glacialis* | 110.359097 | 31.491572 | 554 |
| *Parnassius glacialis* | 118.533622 | 32.0762 | 474 |
| *Parnassius glacialis* | 119.0812 | 32.130051 | 476 |
| *Parnassius glacialis* | 119.454751 | 30.351713 | 607 |
| *Parnassius glacialis* | 107.776701 | 33.838738 | 342 |
| *Parnassius glacialis* | 104.770584 | 32.576596 | 388 |
| *Parnassius glacialis* | 110.929586 | 31.061098 | 569 |
| *Parnassius glacialis* | 119.440303 | 34.708167 | 506 |
| *Parnassius glacialis* | 119.318542 | 31.788155 | 459 |
| *Parnassius glacialis* | 98.55085 | 39.20938 | 267 |
| *Parnassius glacialis* | 104.690707 | 32.947663 | 348 |
| *Parnassius glacialis* | 104.376336 | 33.792735 | 313 |
| *Parnassius glacialis* | 103.845123 | 36.065368 | 184 |
| *Parnassius glacialis* | 104.89685 | 34.72767 | 281 |
| *Parnassius glacialis* | 119.4 | 34.71 | 526 |
| *Parnassius glacialis* | 115.77 | 31.17 | 637 |
| *Parnassius glacialis* | 107.4 | 33.73 | 330 |
| *Parnassius glacialis* | 111.66 | 33.76 | 345 |
| *Parnassius glacialis* | 105.68 | 34.85 | 283 |
| *Parnassius glacialis* | 118.29 | 32.28 | 462 |
| *Parnassius glacialis* | 110.75 | 32.62 | 414 |
| *Parnassius glacialis* | 121.73 | 37.28 | 440 |
| *Parnassius glacialis* | 110.35 | 31.52 | 551 |
| *Parnassius glacialis* | 113.05 | 34.49 | 358 |
| *Parnassius glacialis* | 102.890566 | 34.989612 | 301 |
| *Parnassius glacialis* | 103.176227 | 34.140637 | 316 |
| *Parnassius glacialis* | 103.216053 | 34.063075 | 306 |
| *Parnassius glacialis* | 110.388 | 31.442 | 541 |
| *Parnassius glacialis* | 109.038 | 31.816 | 486 |

**Table S18**. BIO4 values at the 45 occurrence points under current climate conditions.

| **species** | **Longitude** | **Latitude** | **RASTERVALU** |
| --- | --- | --- | --- |
| *Parnassius glacialis* | 118.61113 | 32.111263 | 913.8155518 |
| *Parnassius glacialis* | 118.77778 | 32.06167 | 926.0460205 |
| *Parnassius glacialis* | 120.37194 | 36.09861 | 953.602478 |
| *Parnassius glacialis* | 114.203869 | 31.397749 | 882.8599854 |
| *Parnassius glacialis* | 114.273278 | 31.203545 | 883.130188 |
| *Parnassius glacialis* | 118.562515 | 32.058987 | 913.4574585 |
| *Parnassius glacialis* | 107.789089 | 34.047719 | 824.6483765 |
| *Parnassius glacialis* | 119.081734 | 32.109303 | 919.2225952 |
| *Parnassius glacialis* | 118.833382 | 32.082012 | 919.480835 |
| *Parnassius glacialis* | 119.122328 | 32.118143 | 917.6273804 |
| *Parnassius glacialis* | 119.315865 | 31.795311 | 913.7791748 |
| *Parnassius glacialis* | 117.11289 | 36.241031 | 1003.034241 |
| *Parnassius glacialis* | 108.445445 | 33.439968 | 789.4684448 |
| *Parnassius glacialis* | 108.45 | 33.9 | 804.239563 |
| *Parnassius glacialis* | 119.990294 | 31.813248 | 920.4000244 |
| *Parnassius glacialis* | 118.822224 | 32.06851 | 927.2305908 |
| *Parnassius glacialis* | 110.359097 | 31.491572 | 771.8002319 |
| *Parnassius glacialis* | 118.533622 | 32.0762 | 913.1762695 |
| *Parnassius glacialis* | 119.0812 | 32.130051 | 919.7434082 |
| *Parnassius glacialis* | 119.454751 | 30.351713 | 820.7078857 |
| *Parnassius glacialis* | 107.776701 | 33.838738 | 801.5464478 |
| *Parnassius glacialis* | 104.770584 | 32.576596 | 727.0767822 |
| *Parnassius glacialis* | 110.929586 | 31.061098 | 779.0964966 |
| *Parnassius glacialis* | 119.440303 | 34.708167 | 932.8539429 |
| *Parnassius glacialis* | 119.318542 | 31.788155 | 912.8775024 |
| *Parnassius glacialis* | 98.55085 | 39.20938 | 918.3641357 |
| *Parnassius glacialis* | 104.690707 | 32.947663 | 742.0490723 |
| *Parnassius glacialis* | 104.376336 | 33.792735 | 760.9130859 |
| *Parnassius glacialis* | 103.845123 | 36.065368 | 1001.46814 |
| *Parnassius glacialis* | 104.89685 | 34.72767 | 870.8165283 |
| *Parnassius glacialis* | 119.4 | 34.71 | 934.0891724 |
| *Parnassius glacialis* | 115.77 | 31.17 | 866.8796387 |
| *Parnassius glacialis* | 107.4 | 33.73 | 799.0166626 |
| *Parnassius glacialis* | 111.66 | 33.76 | 881.3399048 |
| *Parnassius glacialis* | 105.68 | 34.85 | 887.3531494 |
| *Parnassius glacialis* | 118.29 | 32.28 | 915.1399536 |
| *Parnassius glacialis* | 110.75 | 32.62 | 877.6470947 |
| *Parnassius glacialis* | 121.73 | 37.28 | 938.2510986 |
| *Parnassius glacialis* | 110.35 | 31.52 | 773.317688 |
| *Parnassius glacialis* | 113.05 | 34.49 | 912.0151978 |
| *Parnassius glacialis* | 102.890566 | 34.989612 | 798.9486084 |
| *Parnassius glacialis* | 103.176227 | 34.140637 | 747.1566162 |
| *Parnassius glacialis* | 103.216053 | 34.063075 | 775.4307861 |
| *Parnassius glacialis* | 110.388 | 31.442 | 784.0361328 |
| *Parnassius glacialis* | 109.038 | 31.816 | 781.4743652 |

**Table S19.** Elevation values at the 45 occurrence points under current climate conditions.

| **species** | **Longitude** | **Latitude** | **RASTERVALU** |
| --- | --- | --- | --- |
| *Parnassius glacialis* | 118.61113 | 32.111263 | 119 |
| *Parnassius glacialis* | 118.77778 | 32.06167 | 17 |
| *Parnassius glacialis* | 120.37194 | 36.09861 | 25 |
| *Parnassius glacialis* | 114.203869 | 31.397749 | 154 |
| *Parnassius glacialis* | 114.273278 | 31.203545 | 208 |
| *Parnassius glacialis* | 118.562515 | 32.058987 | 134 |
| *Parnassius glacialis* | 107.789089 | 34.047719 | 1775 |
| *Parnassius glacialis* | 119.081734 | 32.109303 | 76 |
| *Parnassius glacialis* | 118.833382 | 32.082012 | 111 |
| *Parnassius glacialis* | 119.122328 | 32.118143 | 125 |
| *Parnassius glacialis* | 119.315865 | 31.795311 | 95 |
| *Parnassius glacialis* | 117.11289 | 36.241031 | 634 |
| *Parnassius glacialis* | 108.445445 | 33.439968 | 1762 |
| *Parnassius glacialis* | 108.45 | 33.9 | 2128 |
| *Parnassius glacialis* | 119.990294 | 31.813248 | 5 |
| *Parnassius glacialis* | 118.822224 | 32.06851 | 34 |
| *Parnassius glacialis* | 110.359097 | 31.491572 | 2036 |
| *Parnassius glacialis* | 118.533622 | 32.0762 | 157 |
| *Parnassius glacialis* | 119.0812 | 32.130051 | 94 |
| *Parnassius glacialis* | 119.454751 | 30.351713 | 923 |
| *Parnassius glacialis* | 107.776701 | 33.838738 | 1841 |
| *Parnassius glacialis* | 104.770584 | 32.576596 | 1766 |
| *Parnassius glacialis* | 110.929586 | 31.061098 | 1520 |
| *Parnassius glacialis* | 119.440303 | 34.708167 | 55 |
| *Parnassius glacialis* | 119.318542 | 31.788155 | 90 |
| *Parnassius glacialis* | 98.55085 | 39.20938 | 4893 |
| *Parnassius glacialis* | 104.690707 | 32.947663 | 1311 |
| *Parnassius glacialis* | 104.376336 | 33.792735 | 2443 |
| *Parnassius glacialis* | 103.845123 | 36.065368 | 1522 |
| *Parnassius glacialis* | 104.89685 | 34.72767 | 1576 |
| *Parnassius glacialis* | 119.4 | 34.71 | 205 |
| *Parnassius glacialis* | 115.77 | 31.17 | 645 |
| *Parnassius glacialis* | 107.4 | 33.73 | 1367 |
| *Parnassius glacialis* | 111.66 | 33.76 | 836 |
| *Parnassius glacialis* | 105.68 | 34.85 | 1346 |
| *Parnassius glacialis* | 118.29 | 32.28 | 138 |
| *Parnassius glacialis* | 110.75 | 32.62 | 365 |
| *Parnassius glacialis* | 121.73 | 37.28 | 326 |
| *Parnassius glacialis* | 110.35 | 31.52 | 2073 |
| *Parnassius glacialis* | 113.05 | 34.49 | 632 |
| *Parnassius glacialis* | 102.890566 | 34.989612 | 2954 |
| *Parnassius glacialis* | 103.176227 | 34.140637 | 2914 |
| *Parnassius glacialis* | 103.216053 | 34.063075 | 2603 |
| *Parnassius glacialis* | 110.388 | 31.442 | 1628 |
| *Parnassius glacialis* | 109.038 | 31.816 | 1487 |

**Table S20.** NDVI values at the 45 occurrence points under current climate conditions.

| **species** | **Longitude** | **Latitude** | **RASTERVALU** |
| --- | --- | --- | --- |
| *Parnassius glacialis* | 118.61113 | 32.111263 | 1 |
| *Parnassius glacialis* | 118.77778 | 32.06167 | 1 |
| *Parnassius glacialis* | 120.37194 | 36.09861 | 1 |
| *Parnassius glacialis* | 114.203869 | 31.397749 | 1 |
| *Parnassius glacialis* | 114.273278 | 31.203545 | 1 |
| *Parnassius glacialis* | 118.562515 | 32.058987 | 1 |
| *Parnassius glacialis* | 107.789089 | 34.047719 | 1 |
| *Parnassius glacialis* | 119.081734 | 32.109303 | 1 |
| *Parnassius glacialis* | 118.833382 | 32.082012 | 1 |
| *Parnassius glacialis* | 119.122328 | 32.118143 | 1 |
| *Parnassius glacialis* | 119.315865 | 31.795311 | 1 |
| *Parnassius glacialis* | 117.11289 | 36.241031 | 1 |
| *Parnassius glacialis* | 108.445445 | 33.439968 | 1 |
| *Parnassius glacialis* | 108.45 | 33.9 | 1 |
| *Parnassius glacialis* | 119.990294 | 31.813248 | 1 |
| *Parnassius glacialis* | 118.822224 | 32.06851 | 1 |
| *Parnassius glacialis* | 110.359097 | 31.491572 | 1 |
| *Parnassius glacialis* | 118.533622 | 32.0762 | 1 |
| *Parnassius glacialis* | 119.0812 | 32.130051 | 1 |
| *Parnassius glacialis* | 119.454751 | 30.351713 | 1 |
| *Parnassius glacialis* | 107.776701 | 33.838738 | 1 |
| *Parnassius glacialis* | 104.770584 | 32.576596 | 1 |
| *Parnassius glacialis* | 110.929586 | 31.061098 | 1 |
| *Parnassius glacialis* | 119.440303 | 34.708167 | 1 |
| *Parnassius glacialis* | 119.318542 | 31.788155 | 1 |
| *Parnassius glacialis* | 98.55085 | 39.20938 | 0 |
| *Parnassius glacialis* | 104.690707 | 32.947663 | 1 |
| *Parnassius glacialis* | 104.376336 | 33.792735 | 1 |
| *Parnassius glacialis* | 103.845123 | 36.065368 | 0.296099991 |
| *Parnassius glacialis* | 104.89685 | 34.72767 | 1 |
| *Parnassius glacialis* | 119.4 | 34.71 | 1 |
| *Parnassius glacialis* | 115.77 | 31.17 | 1 |
| *Parnassius glacialis* | 107.4 | 33.73 | 1 |
| *Parnassius glacialis* | 111.66 | 33.76 | 1 |
| *Parnassius glacialis* | 105.68 | 34.85 | 1 |
| *Parnassius glacialis* | 118.29 | 32.28 | 1 |
| *Parnassius glacialis* | 110.75 | 32.62 | 1 |
| *Parnassius glacialis* | 121.73 | 37.28 | 1 |
| *Parnassius glacialis* | 110.35 | 31.52 | 1 |
| *Parnassius glacialis* | 113.05 | 34.49 | 1 |
| *Parnassius glacialis* | 102.890566 | 34.989612 | 1 |
| *Parnassius glacialis* | 103.176227 | 34.140637 | 1 |
| *Parnassius glacialis* | 103.216053 | 34.063075 | 1 |
| *Parnassius glacialis* | 110.388 | 31.442 | 1 |
| *Parnassius glacialis* | 109.038 | 31.816 | 1 |

**Table S21**. HPD values at the 45 occurrence points under current climate conditions.

| **species** | **Longitude** | **Latitude** | **RASTERVALU** |
| --- | --- | --- | --- |
| *Parnassius glacialis* | 118.61113 | 32.111263 | 1667.027222 |
| *Parnassius glacialis* | 118.77778 | 32.06167 | 40528.58984 |
| *Parnassius glacialis* | 120.37194 | 36.09861 | 31838.33789 |
| *Parnassius glacialis* | 114.203869 | 31.397749 | 273.3438416 |
| *Parnassius glacialis* | 114.273278 | 31.203545 | 201.4958801 |
| *Parnassius glacialis* | 118.562515 | 32.058987 | 788.9319458 |
| *Parnassius glacialis* | 107.789089 | 34.047719 | 116.3426971 |
| *Parnassius glacialis* | 119.081734 | 32.109303 | 482.4888611 |
| *Parnassius glacialis* | 118.833382 | 32.082012 | 10298.35254 |
| *Parnassius glacialis* | 119.122328 | 32.118143 | 198.1906586 |
| *Parnassius glacialis* | 119.315865 | 31.795311 | 380.903717 |
| *Parnassius glacialis* | 117.11289 | 36.241031 | 1243.287231 |
| *Parnassius glacialis* | 108.445445 | 33.439968 | 34.24414444 |
| *Parnassius glacialis* | 108.45 | 33.9 | 44.66268921 |
| *Parnassius glacialis* | 119.990294 | 31.813248 | 11615.89453 |
| *Parnassius glacialis* | 118.822224 | 32.06851 | 19304.88867 |
| *Parnassius glacialis* | 110.359097 | 31.491572 | 12.66868114 |
| *Parnassius glacialis* | 118.533622 | 32.0762 | 354.6078186 |
| *Parnassius glacialis* | 119.0812 | 32.130051 | 530.9390259 |
| *Parnassius glacialis* | 119.454751 | 30.351713 | 67.81788635 |
| *Parnassius glacialis* | 107.776701 | 33.838738 | 6.312617302 |
| *Parnassius glacialis* | 104.770584 | 32.576596 | 0.396132886 |
| *Parnassius glacialis* | 110.929586 | 31.061098 | 88.48728943 |
| *Parnassius glacialis* | 119.440303 | 34.708167 | 149.7246857 |
| *Parnassius glacialis* | 119.318542 | 31.788155 | 748.0014648 |
| *Parnassius glacialis* | 98.55085 | 39.20938 | 0.395012945 |
| *Parnassius glacialis* | 104.690707 | 32.947663 | 95.73838043 |
| *Parnassius glacialis* | 104.376336 | 33.792735 | 334.469574 |
| *Parnassius glacialis* | 103.845123 | 36.065368 | 10833.77246 |
| *Parnassius glacialis* | 104.89685 | 34.72767 | 542.1843262 |
| *Parnassius glacialis* | 119.4 | 34.71 | 1939.254639 |
| *Parnassius glacialis* | 115.77 | 31.17 | 66.42253113 |
| *Parnassius glacialis* | 107.4 | 33.73 | 9.399969101 |
| *Parnassius glacialis* | 111.66 | 33.76 | 528.8551636 |
| *Parnassius glacialis* | 105.68 | 34.85 | 822.7609253 |
| *Parnassius glacialis* | 118.29 | 32.28 | 467.9928589 |
| *Parnassius glacialis* | 110.75 | 32.62 | 4791.326172 |
| *Parnassius glacialis* | 121.73 | 37.28 | 167.3782806 |
| *Parnassius glacialis* | 110.35 | 31.52 | 12.66868114 |
| *Parnassius glacialis* | 113.05 | 34.49 | 1463.479858 |
| *Parnassius glacialis* | 102.890566 | 34.989612 | 658.2997437 |
| *Parnassius glacialis* | 103.176227 | 34.140637 | 8.432310104 |
| *Parnassius glacialis* | 103.216053 | 34.063075 | 41.48446655 |
| *Parnassius glacialis* | 110.388 | 31.442 | 12.66868019 |
| *Parnassius glacialis* | 109.038 | 31.816 | 32.38143539 |
